# Genome sequencing of *Inonotus obliquus* reveals insights into candidate genes involved in secondary metabolite biosynthesis

**DOI:** 10.1186/s12864-022-08511-x

**Published:** 2022-04-20

**Authors:** Yingce Duan, Haiyan Han, Jianzhao Qi, Jin-ming Gao, Zhichao Xu, Pengchao Wang, Jie Zhang, Chengwei Liu

**Affiliations:** 1 Key Laboratory for Enzyme and Enzyme-Like Material Engineering of Heilongjiang, College of Life Science, Northeast Forestry University, Harbin, 150040 Heilongjiang China; 2 Shaanxi Key Laboratory of Natural Products & Chemical Biology, College of Chemistry & Pharmacy, Northwest A&F University, Yangling, 712100 Shaanxi China

**Keywords:** *Inonotus obliquus*, Genome sequencing, CAZymes, P450, Secondary metabolites

## Abstract

**Background:**

*Inonotus obliquus* is an important edible and medicinal mushroom that was shown to have many pharmacological activities in preclinical trials, including anti-inflammatory, antitumor, immunomodulatory, and antioxidant effects. However, the biosynthesis of these pharmacological components has rarely been reported. The lack of genomic information has hindered further molecular characterization of this mushroom.

**Results:**

In this study, we report the genome of *I. obliquus* using a combined high-throughput Illumina NovaSeq with Oxford Nanopore PromethION sequencing platform. The de novo assembled 38.18 Mb *I. obliquus* genome was determined to harbor 12,525 predicted protein-coding genes, with 81.83% of them having detectable sequence similarities to others available in public databases. Phylogenetic analysis revealed the close evolutionary relationship of *I. obliquus* with *Fomitiporia mediterranea* and *Sanghuangporus baumii* in the Hymenochaetales clade. According to the distribution of reproduction-related genes, we predict that this mushroom possesses a tetrapolar heterothallic reproductive system. The *I. obliquus* genome was found to encode a repertoire of enzymes involved in carbohydrate metabolism, along with 135 cytochrome P450 proteins. The genome annotation revealed genes encoding key enzymes responsible for secondary metabolite biosynthesis, such as polysaccharides, polyketides, and terpenoids. Among them, we found four polyketide synthases and 20 sesquiterpenoid synthases belonging to four more types of cyclization mechanism, as well as 13 putative biosynthesis gene clusters involved in terpenoid synthesis in *I. obliquus*.

**Conclusions:**

To the best of our knowledge, this is the first reported genome of *I. obliquus*; we discussed its genome characteristics and functional annotations in detail and predicted secondary metabolic biosynthesis-related genes, which provides genomic information for future studies on its associated molecular mechanism.

**Supplementary Information:**

The online version contains supplementary material available at 10.1186/s12864-022-08511-x.

## Background

*Inonotus obliquus* (Ach. ex Pers.) Pilát, a wild medicinal mushroom, is mainly distributed in Russia (called Chaga), Scandinavia, Central Europe, and Eastern Europe [1]. This medicinal fungus is enriched in many active chemical components [2] and is used for disease treatment [3] in Russia and northeastern China as a folk remedy. *I. obliquus*, which is parasitic or saprophytic on birch and other trees, is a white rot fungus [4]. Its growth temperature ranges from 25 to 30 °C, with pH 6 [5], and it grows at high latitudes and in cold regions. Accordingly, fruiting body or sclerotium formation in *I. obliquus* is slower and the entire growth cycle is longer, limiting its extensive development and utilization [2, 6].

Many important secondary metabolites can be derived from the mycelia, fruit body, and sclerotium, such as polysaccharides, melanin, phenols, and terpenoids [3]. These compounds of *I. obliquus* have significant pharmaceutical value, such as anti-tumor, anti-inflammatory, anti-oxidant [3], anti-microbial [7], and anti-neuroinflammatory [8]. Research has shown that 150 μg/ml polysaccharide from *I. obliquus*, which can restrain the growth of a hepatoma cell line, exhibits an inhibitory rate similar to that of mitomycin at a dose of 5 μg/ml [9]. Melanin of *I. obliquus* facilitates an increase in the growth of *Bifidobacterium bifidum* 1 by 1.4-fold in comparison to that with ascorbic acid, as a control in the trials, after 24 h of cultivation [10]. The contents and species of triterpenoids from *I. obliquus* are abundant and complex, including trametenolic acid, inotodiol, and betulinic acid and so on. These compounds reduce the viability of human cancer cell lines (IC_50_ value < 5 µM) and have anti-proliferative properties [11]. Despite increasing interest in the active components of *I. obliquus*, to date, very little is known about the molecular and genetic basis of the biosynthetic pathways yielding these components.

In recent years, rapid advancements in technology have gradually led to the analysis of genomes of many medicinal mushrooms, like *Ganoderma lucidum* [12], *Antrodia cinnamomea* [13], *Hericium erinaceus* [14], *Sanghuangporus baumii* [15], and *Wolfiporia cocos* [16]. Regarding the molecular mechanisms of *I. obliquus* secondary metabolism, transcriptome analysis of Chaga cultured with different betulin sources unveiled the genes responsible for the terpenoid pathways [17]. The farnesyl pyrophosphate synthase gene [18] and squalene synthase [19] from *I. obliquus* were cloned and characterized for the biosynthesis of sterols and triterpenes.

Here, we report the genome sequence of *I. obliquus* based on single-molecule real-time reads from the Nanopore platform and that combined with an Illumina sequencing strategy*.* First, characterization analysis of the *I. obliquus* genome included gene content and genome structure. Second, we identified functional genes and gene clusters involved in secondary metabolite biosynthesis, such as polysaccharides, melanin, and terpenoids. Third, we performed a classification analysis of the P450 gene family involved in secondary metabolism and biosynthesis.

## Results

### Genome sequence assembly and annotation

**Fig. 1:**
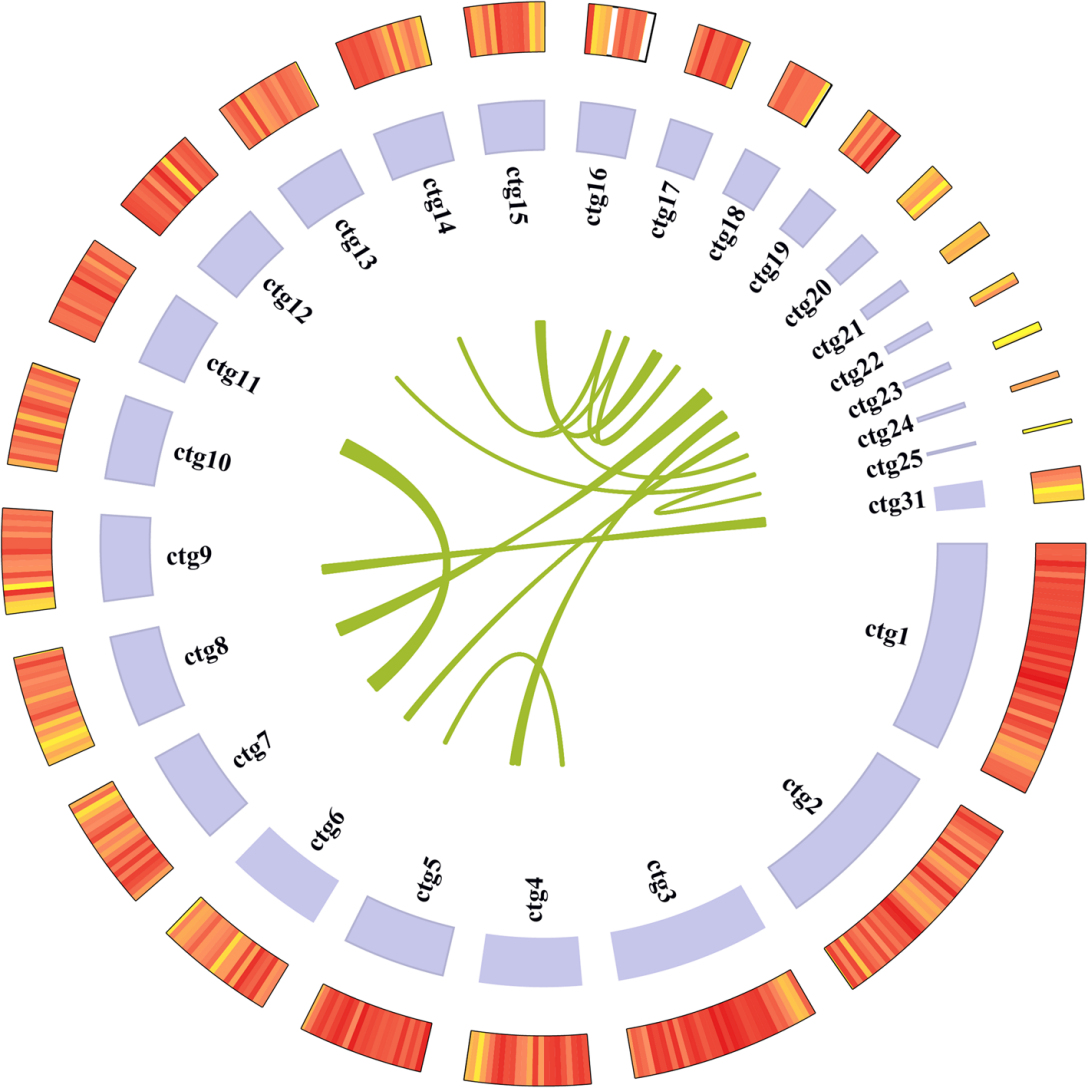
Genome diagram of *Inonotus obliquus* genome. The outermost circle is gene density, and the innermost circle is collinearity analysis. The middle circle is the length (bp) of each contig. Remaining contigs are not displayed because there were no gene annotations and their gene sizes were small

In total, 37,218,262 clean reads were generated, and the total number of bases was 5,582,739,300 (Table S1-2). The genome size was 38.18 Mb. This consisted of 31 contigs with an N50 of 1.88 Mb and 47.56% GC content (Fig. 1). The mapping rates of Illumina NovaSeq sequencing data have exceeded 99%, and BUSCO assessment indicated that assembly completeness was close to 90%. Our results indicated that genome assembly was of good quality (Table S3-4). We predicted 12,525 protein-coding genes, and 80% of the genes were annotated. The average CDS sequence length was 1,296 bp, and the longest contig length was 4.38 Mb (Table 1). On average, each predicted gene contained 7.19 exons. Genes typically contained small exons (average, 180.37 bp) and introns (average, 72.13 bp), similar to that in other basidiomycetes. For non-coding RNA, 88 tRNAs, 78 rRNAs, 14 snRNAs, and one sRNA were predicted. The number of long terminal repeats was 1,286, occupying 3.9% of the whole genome, the number of DNA transposons was 732, occupying 0.9% of the whole genome, the number of simple repetitions was 5,401, occupying 0.59% of the whole genome, and the number of satellite repetitions was 11 (Table S5-7). To obtain comprehensive gene function information, 12,525 non-redundant genes were subjected to similarity analysis based on several public databases. Most of these genes were mapped using the Nr database, specifically 10,249 genes/81.83%, followed by Pfam (7,956 genes/63.52%), Interproscan (7,924 genes/63.27%), Uniprot (5,468 genes/45.09%), Gene ontology (GO; 5,602 genes/44.73%), Kyoto Encyclopedia of Genes and Genomes (KEGG; 4,121 genes/32.90%), Refseq (3,945 genes/31.50%), Pathway (2,509 genes/20.03%), and Clusters of Orthologous Groups (COG; 1,112 genes/8.88%) (Table 1).

**Table 1:** *Inonotus obliquus* genome assembly and functional annotation

Item	Value	Item	Number	Percentage
Total_length(bp)	38,181,337	All	12,525	100%
Contigs	31	Nr	10,249	81.83%
GC_content(%)	47.56	Pfam	7,956	63.52%
N50(bp)	1,971,511	Interproscan	7,924	63.27%
N90(bp)	915,121	Uniprot	5,648	45.09%
Average(bp)	1,193,166.78	GO	5,602	44.73%
Median(bp)	1,027,062.5	KEGG	4,121	32.90%
Min(bp)	2774	Refseq	3,945	31.50%
Max(bp)	4,380,421	Pathway	2,509	20.03%
Total number of genes	12,525	COG	1,112	8.88%

**Fig. 2:**
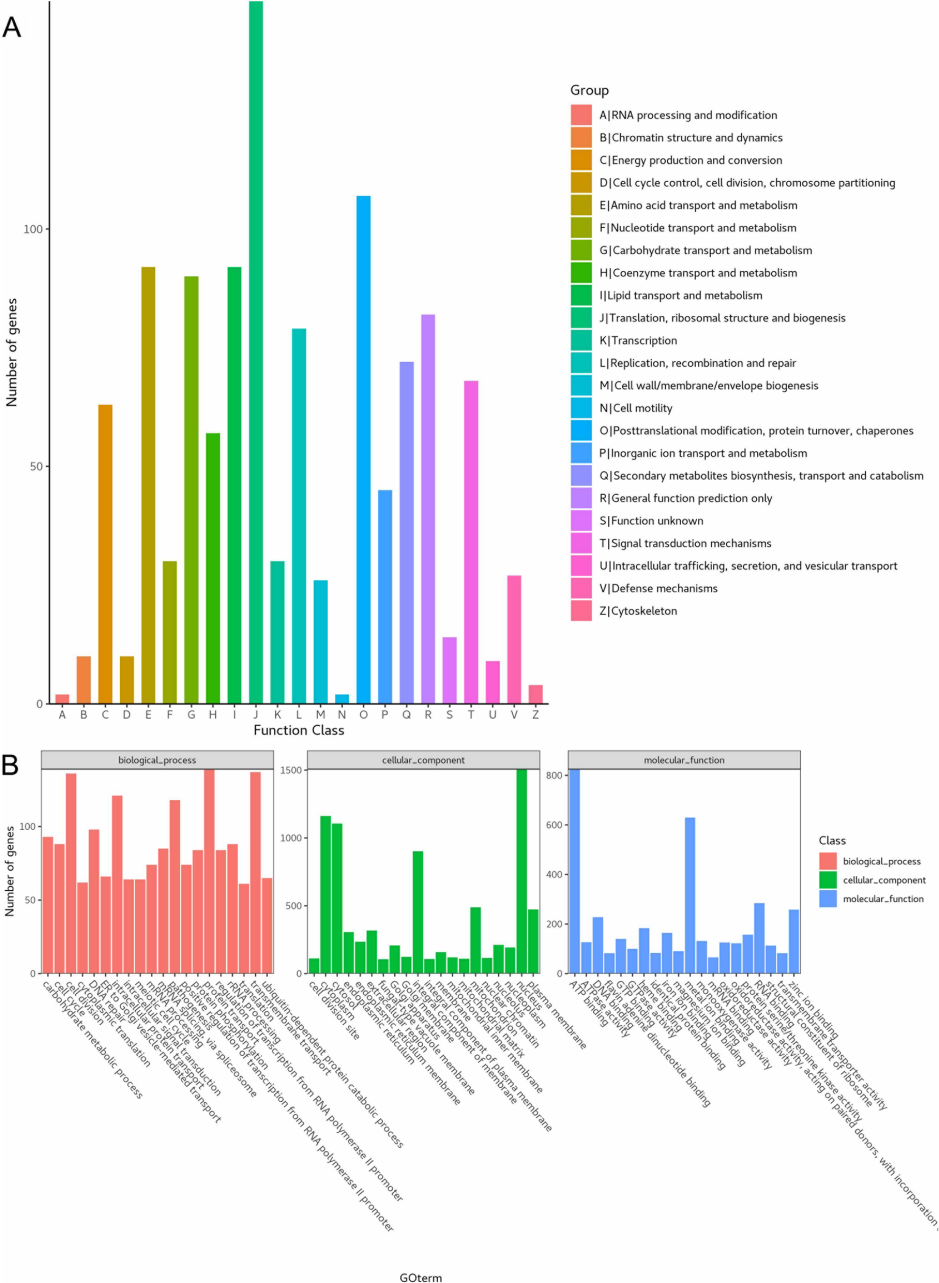
Genomic functional annotation of *Inonotus obliquus*. **A** Cluster of Orthologous Groups of proteins (COG), **B** Gene Ontology (GO)

**Fig. 3:**
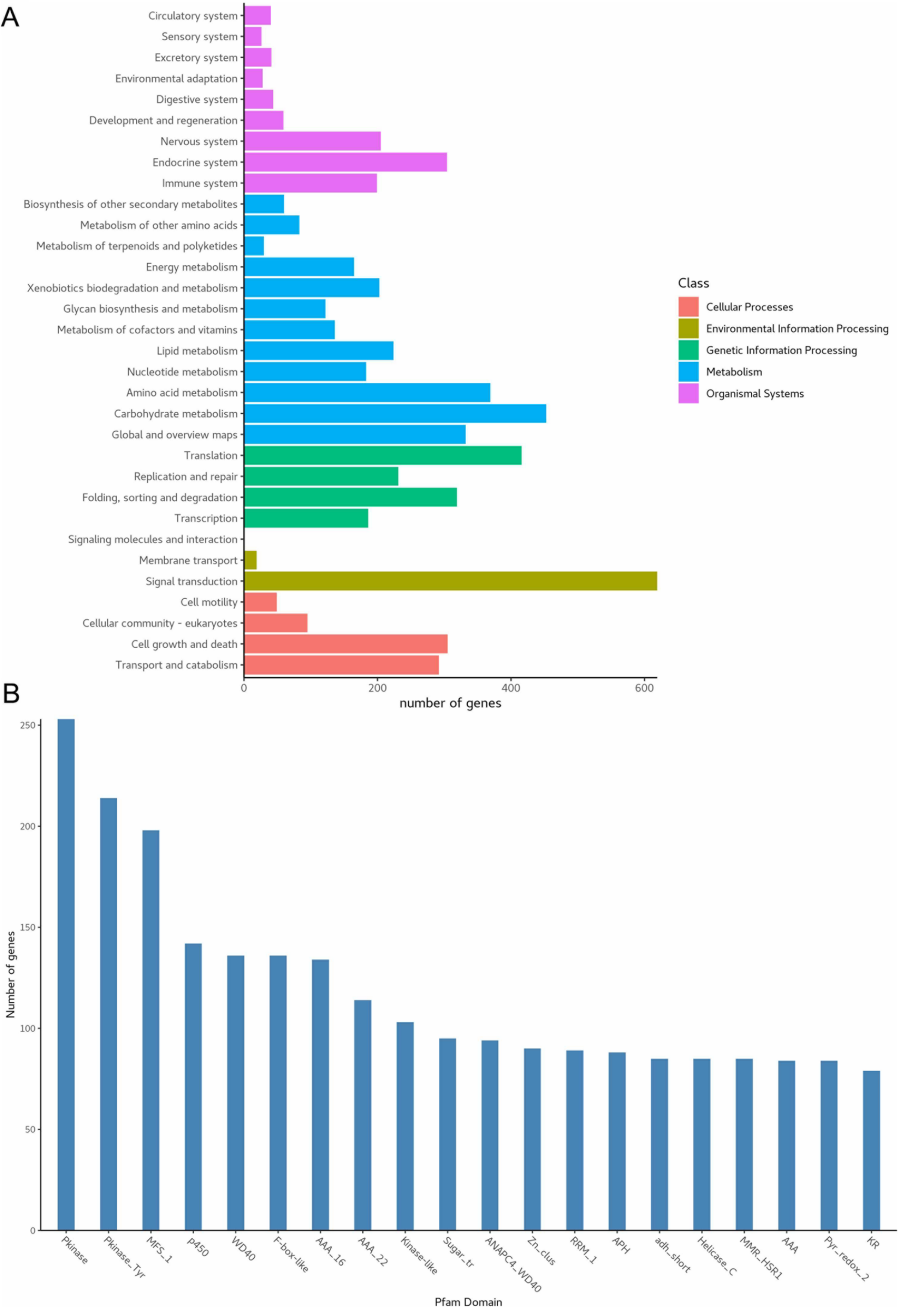
Genomic functional annotation of *Inonotus obliquus*. **A** Kyoto Encyclopedia of Genes and Genomes (KEGG), **B** Pfam Domain

According to the COG database, “translation, ribosomal structure and biogenesis” was associated with the most genes (148). This was followed by “posttranslational modification, protein turnover, chaperones”, “amino acid transport and metabolism”, and “lipid transport and metabolism” as the most gene-rich classes in the COG groupings (Fig. 2A). These findings suggest the presence of an enriched and varied array of protein and lipid metabolism functions that enable higher energy conversion efficiency. GO annotation resulted in the nucleus (1,507), cytoplasm (1,167), and cytosol (1,105) from the cellular component category, protein transport (139) from biological processes, and ATP binding (825) and metal ion binding (630) from molecular functions (Fig. 2B). These results show that the most abundant genes in the genome are the metabolism of genetic material and energy. The KEGG functional classification showed signal transduction (619), carbohydrate metabolism (453), and translation (416) (Fig. 3A). *I. obliquus* is a wild strain, in which many metabolic genes are involved in signal transduction, indicating a high degree of adaptability to the environment. In addition, we identified 253 Pkinase, 198 MFS, and 136 WD40 genes in the pfam domain of the *I. obliquus* genome (Fig. 3B).

### Phylogenetic analysis of other fungal GENOMES

**Fig. 4:**
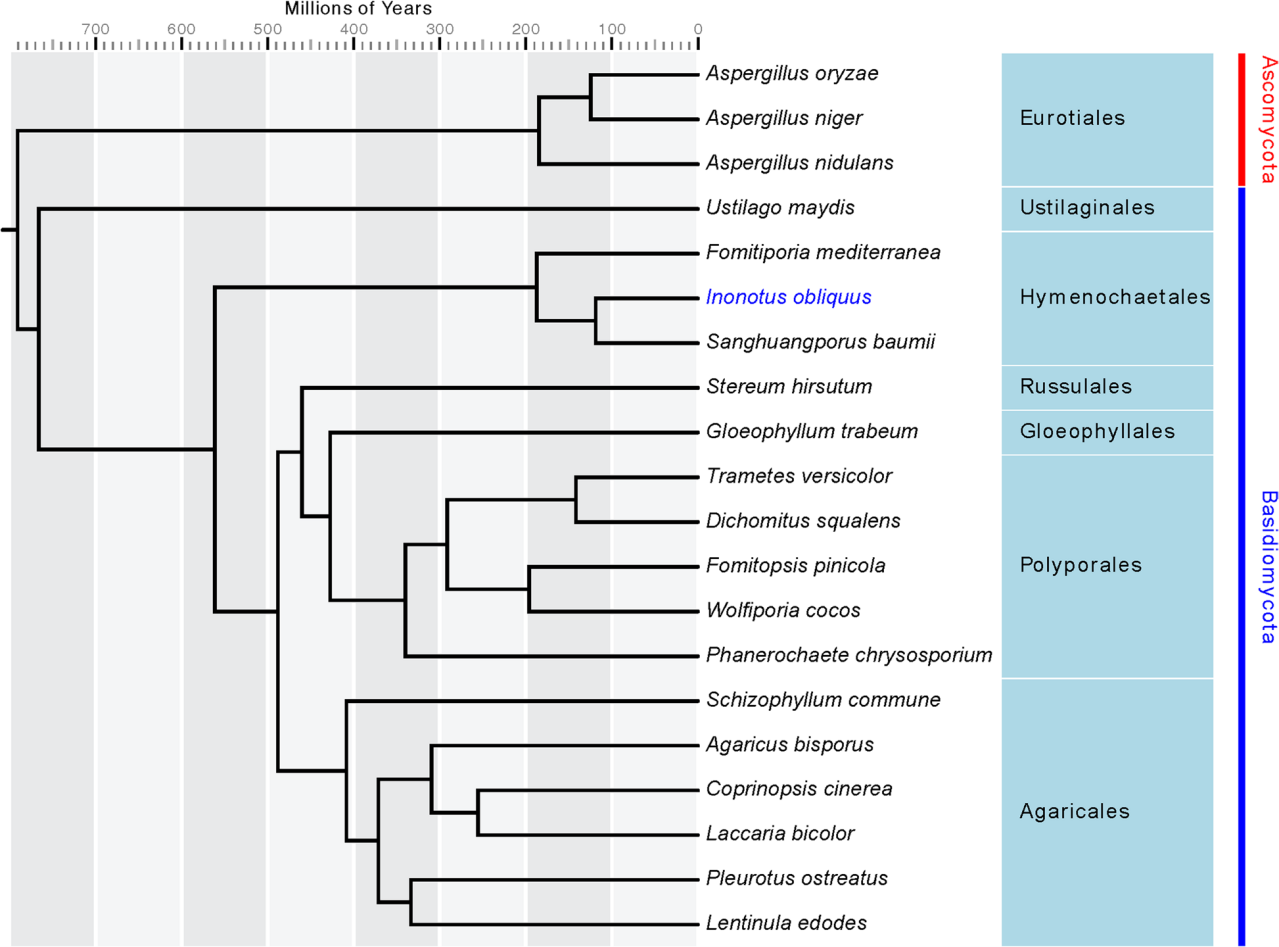
Phylogenetic analysis of 20 fungal species based on 699 single-copy orthologous genes

To investigate the evolutionary history and classification status of the *I. obliquus* genome, we identified a total of 18,571 homologous gene families, among which *I. obliquus* had 7,154 families. The *I. obliquus* genome was found to contain 407 specific families. In total, 699 single-copy orthologous genes were used for phylogenetic tree construction. We found that these 20 fungal species were distributed on two branches, Basidiomycetes and Ascomycetes (Table S8). The branch of Basidiomycetes was further divided into six subgroups, which corresponded to six orders, Agaricales, Polyporales, Gloeophyllales, Russulales, Hymenochaetales, and Ustilaginales. The phylogenetic tree showed that the estimated divergence time between the *I. obliquus* lineage and *F. mediterranea* lineage was approximately 195 million years ago (Mya) and that from the *S. baumii* lineage was approximately 100 Mya. These two species belong to Hymenochaetales. The relationship was distant between *I. obliquus* and *W. cocos*, *D. squalens*, or *T. versicolor*, species from the Polyporales order. *I. obliquus*, *S. hirsutum*, and *S. commune* were determined to have the same ancestor, and the estimated divergence time was approximately 570 Mya (Fig. 4).

### Carbohydrate genes

In this study, 380 genes encoding carbohydrate-active enzymes (CAZymes) were found. These included 192 glycoside hydrolases (GHs), 75 auxiliary activities (AAs), 66 glycosyltransferases (GTs), 24 carbohydrate esterases (CEs), 11 carbohydrate-binding modules (CBMs), and 13 polysaccharide lyases (PLs) (Table 2). As white rot fungi, because of strong lignocellulose-degradation activity, carbohydrate genes of *I. obliquus* exceeded those of *G. trabeum*, *F. pinicola*, and *W. cocos*, brown rot fungi, as well as the symbiotic fungus *L. bicolor*. Straw rot *A. bisporus* and *C. cinereus* were determined to have similar or even higher quantities of the *I. obliquus* carbohydrate gene. Compared with *I. obliquus*, the white rot fungi *S. hirsutum*, *T. versicolor*, *P. ostreatus*, and *L. edodes* had more carbohydrate genes. In the *I. obliquus* genome, GHs were distributed across 46 families. Cellulose and hemicellulose-degrading enzymes mostly belonged to GH1, GH3, and GH6 families. AAs mainly included AA1–3, AA5–AA9, and AA14 (nine families). Lignin-degrading enzymes mainly belonged to the AA1 and AA2 families in the genome. GTs contained 29 families, including eight chitin synthetases belonging to the GT2 family. CBMs were classified as CBM1, CBM5, CBM13, CBM20, and CBM21 (five families). CEs were classified as CE1, CE4, CE8–9, CE12, and CE15–17 (eight families). PLs were mainly distributed in five families, including PL1, PL8, PL14, PL35, and PL38.

**Table 2:** Gene distribution of different fungi based on the six major modules of CAZymes

CAZy	Total	GH	AA	GT	CBM	CE	PL
*Inonotus obliquus*	380	199	76	66	11	24	13
*Sanghuangporus baumii*	329	175	63	61	5	18	9
*Fomitiporia mediterranea*	387	197	91	66	5	22	8
*Coprinopsis cinerea*	469	188	129	72	21	47	18
*Schizophyllum commune*	469	247	85	71	11	37	19
*Gloeophyllum trabeum*	350	199	57	62	5	19	11
*Dichomitus squalens*	433	218	102	65	9	28	15
*Phanerochaete chrysosporium*	383	183	102	66	10	21	8
*Laccaria bicolor*	311	151	53	73	9	9	7
*Agaricus bisporus*	370	172	94	52	11	33	12
*Pleurotus ostreatus*	505	226	139	65	29	27	26
*Stereum hirsutum*	518	274	127	68	7	29	19
*Trametes versicolor*	441	223	106	78	6	19	13
*Wolfiporia cocos*	269	146	42	63	2	13	4
*Lentinula edodes*	449	241	86	71	14	32	11
*Fomitopsis pinicola*	350	201	56	65	5	20	5
*Ustilago maydis*	215	106	26	63	1	17	2
*Aspergillus nidulans*	486	259	89	81	20	32	23
*Aspergillus niger*	503	253	105	102	17	30	10
*Aspergillus oryzae*	538	289	96	93	17	31	26

### Mating genes

Mating type recognition plays a role in the genetics and breeding of mushrooms, determining the propagating system, fruiting body, and gamete quality. Homothallism is selfing fertility and heterothallism is a hybrid conception. Homothallism can be divided into biopolar and tetrapolar. *L. edodes* and *G. lucidum* are tetrapolar, whereas *Cordyceps militaris* and *W. cocos* are biopolar [20]. The biopolar homothallism sex system comprises a single factor; every monokaryon has only one mating type, and only different types of monokaryons can mate with a heterokaryon at the time of sexual reproduction. In a tetrapolar mating system, genes in mate A encode homeodomain (HD) transcription factors, and those of mate B encode the pheromone receptor and pheromone precursor genes. Mate A controls hook cell formation, nucleus pairing, hyphal cell fusion, and lock-cell formation. Mate B controls septal dissolution, nuclear migration, and lock-cell fusion with the subapical cells. Only when A and B mating types are different between the two monokaryons can mating be successful. The mating type of *I. obliquus* has not been reported to date*.*

**Fig. 5:**
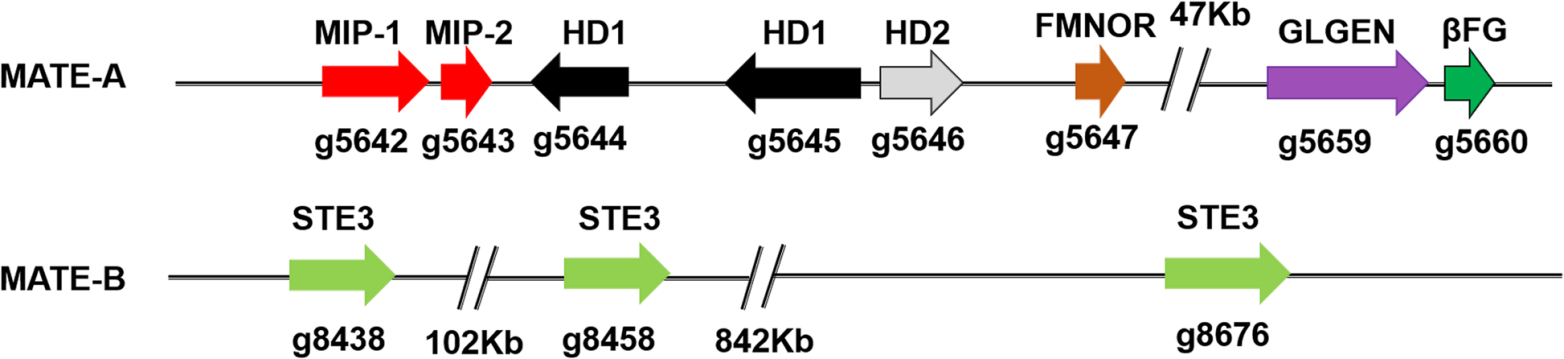
Distribution of mating type genes in *Inonotus obliquus*. *MIP* Mitochondrial intermediate peptidase, *HD1* Homeodomain protein 1, HD2 Homeodomain protein 2, *FMNOR* FMN-linked oxidoreductase, *GLGEN* Glycosyltransferase family 8 protein, *βFG* Beta-flanking gene; STE, STE3-like pheromone receptor

We found HD-encoding protein type-related genes in the genome; HD1 was g5645, HD2 was g5644, and MIP genes were g5642 and g5643 (Table S9). The positional continuity of these four genes was located in contig 1. Pheromone receptors included g8676, g8458, and g8438, located in contig 15 (Fig. 5). The B mating type locus was not found to be linked with homeodomain transcription factors (A mating type locus) in the tetrapolar region. According to the genome information, two mating types were not in the same contig, and we predicted that the mate type of *I. obliquus* is likely tetrapolar, but determination of the real mating type requires further testing and verification.

### Polysaccharide biosynthesis

*I. obliquus* polysaccharides own specially biological activities, with monose components mainly comprising mannose, glucose, galactose, xylose, rhamnose, and arabinose [21]. The main source of fungal polysaccharides is the cell wall of mycelia. Phosphoglucomutase, β-glucan synthase, and UDP-glucose 6-dehydrogenase are key enzymes involved in polysaccharide biosynthesis. As *G. lucidum* polysaccharides involved in biosynthetic pathway correlational studies, we found 18 genes involved in polysaccharide biosynthesis, including one phosphoglucomutase, one UDP-glucose-6-dehydrogenase, one UDP-glucose 4-epimerase and two 1,3-beta-glucan synthases, as well as five genes encoding beta-glucan synthesis-associated proteins (Table S10). Compare with other species of medicinal mushrooms (*S. baumii*), *I. obliquus* possesses similar numbers of genes related to polysaccharide biosynthesis.

### Polyketide biosynthesis

**Fig. 6:**
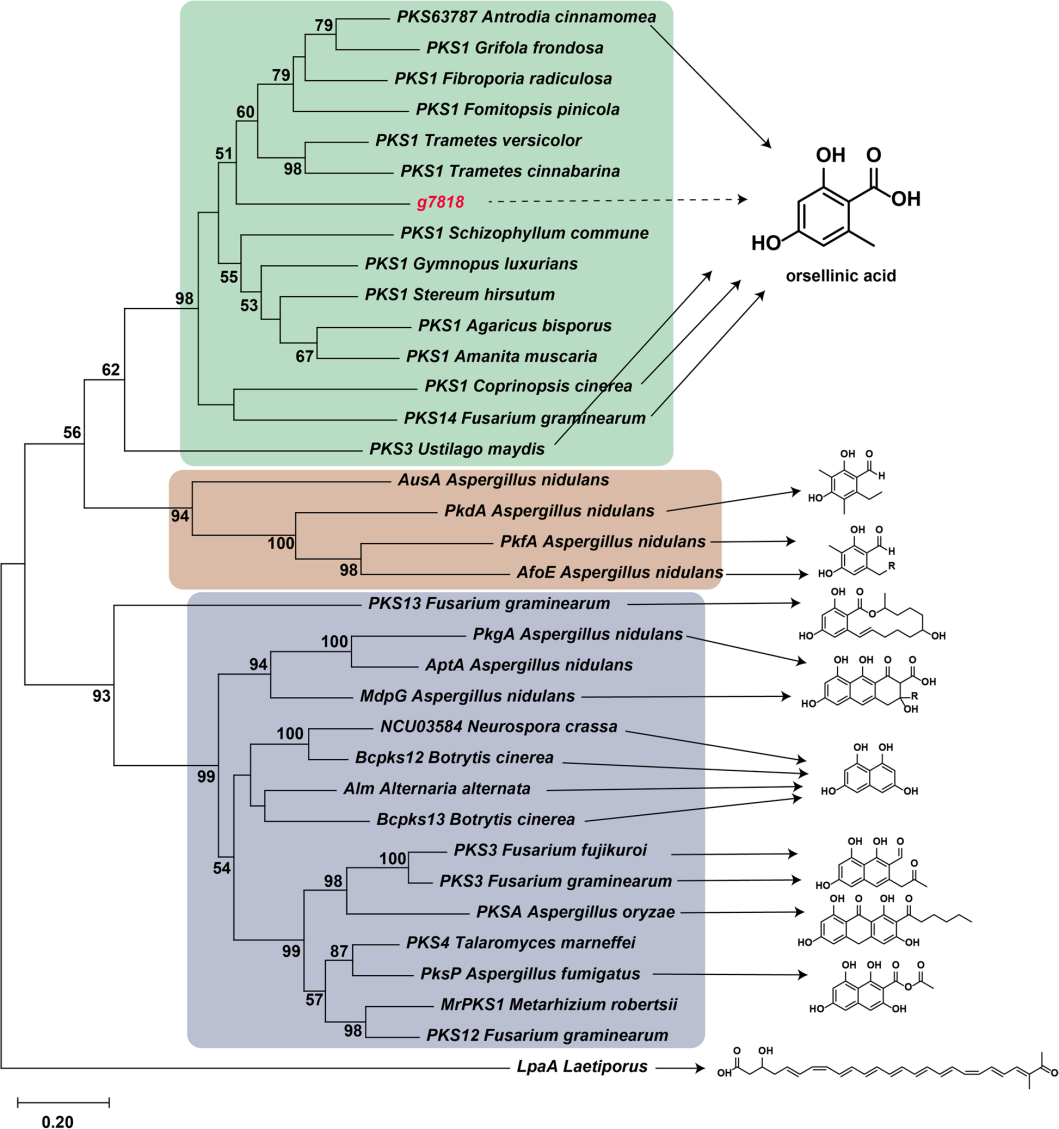
Phylogenetic tree of different functional PKS enzymes constructed by maximum likelihood analysis of the keto-synthase (KS) domain amino acid sequences

Polyketides represent a large group of structurally diverse secondary metabolites, including tetracycline, erythromycin, lovastatin. In addition, various pigments, polyphenols, and a plethora of mycotoxins, such as the aflatoxins and fumonisins, are produced via the polyketide pathway [22]. Polyketide synthase is usually composed of multiple modules (such as acyl transferase (AT), acyl carrier protein domain (ACP)), which then function to form polyketides, of which, the more important module is the ketosynthase domain (KS) modules, catalyzing the actual condensation step. Compared with that in different fungal species, we found that the number of PKSs in the basidiomycete genome was much less than that in ascomycetes, and the number in basidiomycetes is basically fewer than 10, whereas PKSs often appeared to be combined with non-ribosomal peptide synthetase (NRPS). We found that there were one PKS, two PKS-NRPSs, and four NRPSs in *I. obliquus* (Table S11). There are various types of polyketides, mainly occurring through the formation of different skeletal structures mediated by PKS. At present, there are relatively few studies on PKS in basidiomycetes, mainly including *Coprinopsis cinerea* [23], *Antrodia cinnamomea* [24], *Laetiporus* [25], and *Ustilago maydis* [26]. These can synthesize different skeleton compounds, such as orsellinic acid,  polyenes or polyketide-based melanin with a distinct biosynthetic route [26]. Phylogenetic tree analysis of different PKSs (KS domain) [24] showed that g7818 might be an orsellinic acid synthesis gene involved in the formation of orsellinic acid in *I. obliquus* (Fig. 6).

### Terpenoid biosynthesis

Terpenoids are one of the main secondary metabolites in *I. obliquus*, and 86 bioactive metabolites have been reported to date [27]. Most of these major terpenoid synthesis sources are mainly derived from the mevalonate pathway, which consists of 11 enzymes and 16 genes, including acetyl-CoA acyltransferase, based on four coding genes, mevalonate kinase and farnesyl diphosphate (FPP), which are encoded by two genes, and other enzymes encoded by a single-copy gene (Table S13). In this study, we found 19 gene clusters of secondary metabolites by AntiSMASH fungal 6.0.0, with different contigs and 13 terpenoids synthesis-related gene clusters distributed. There were 22 genes related to terpenoid synthase in the *I. obliquus* genome, including 20 sesquiterpene synthases (STSs), one lanosterol synthase, and one geranylgeranyl diphosphate synthase.

**Fig. 7:**
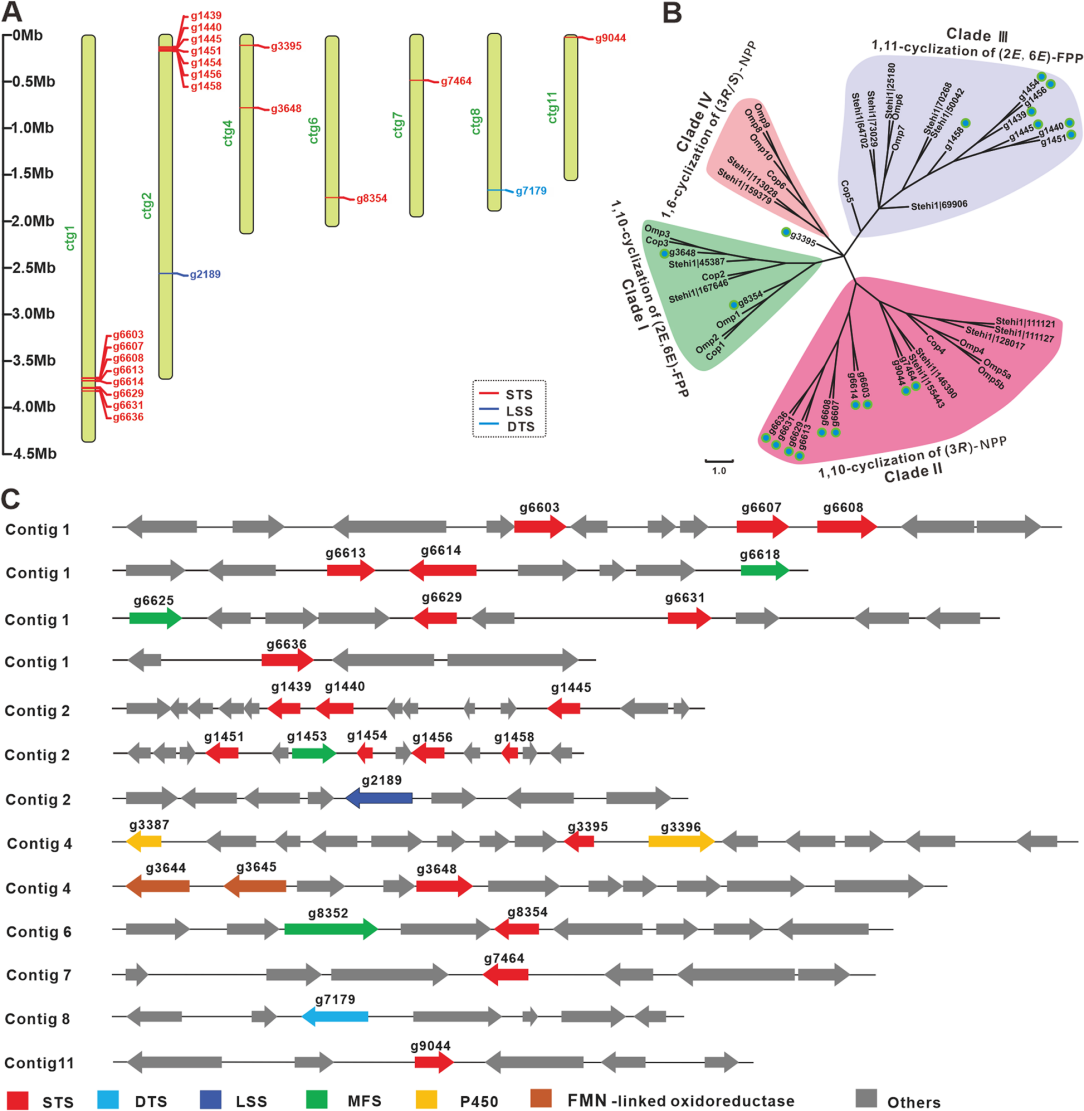
Terpene synthases in *Inonotus obliquus.* **a** Distribution of terpene synthases on the different contigs, **b** phylogenetic analysis of sesquiterpene synthase (STS) homologues, **c** gene clusters in *I. obliquus*. DTS is diterpene synthase, and LSS is lanosterol synthase

According to the same conserved domain, 20 genes were determined to be probably involved in sesquiterpene synthase. We used other known sesquiterpene synthases such as those of *Omphalotus olearius* [28], *S. hirsutum* [29], and *C. cinereus* [30] as identification criteria to identify the types of sesquiterpene synthases in *I. obliquus*. The 20 sesquiterpene synthases were divided into three clades. There were 10 sesquiterpene synthases belonging to Clade II, seven sesquiterpene synthases belonging to Clade III, two sesquiterpene synthases belonging to Clade I, and one was not assigned (Fig. 7B). Clade II consisted of enzymes that shared a 1,10-cyclization of (3R)-nerolidyl diphosphate mechanism, producing sesquiterpenes derived from a *Z, E*-germacradienyl cation. Clade III consists of enzymes believed to share a common 1,11-cyclization of the (2E,6E)-FPP mechanism, producing the trans-humulyl cation. Clade I consisted of enzymes that utilize a 1,10-cyclization of (2E,6E)-FPP to produce sesquiterpenes derived from a *E,E*-germacradienyl cation [30]. It can be seen from the position of the contigs in which the genes were located that sesquiterpene synthases were almost all concentrated at the two ends of the contig (Fig. 7A). This distribution phenomenon was consistent with the distribution of genes related to secondary metabolism. In the genes surrounding sesquiterpene synthase, we found that membrane transporters and cytochrome P450 were related to sesquiterpene synthesis (Fig. 7C).

### Cytochrome P450 monooxygenase (CYP) family analysis

**Fig. 8:**
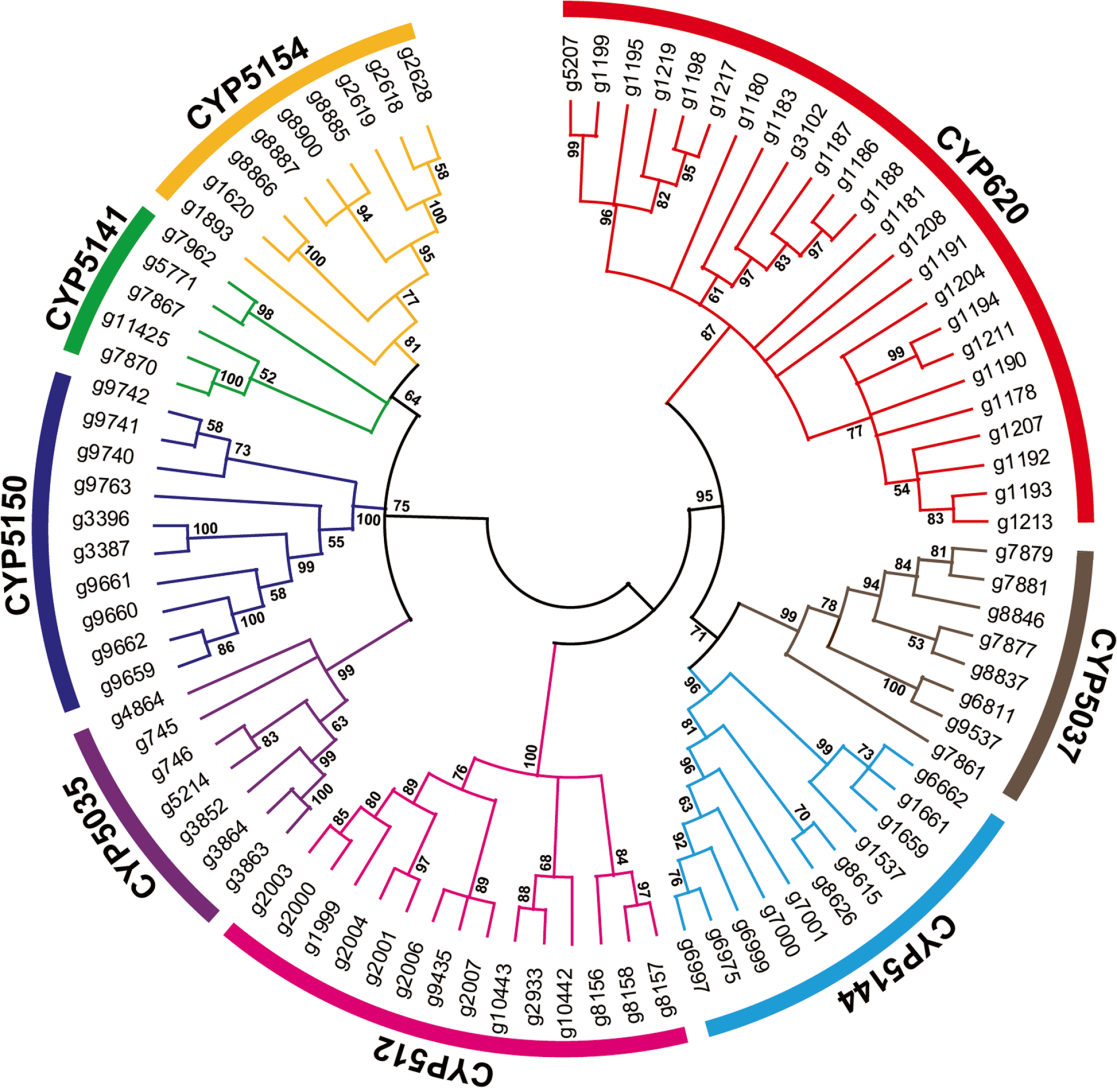
Phylogenetic tree composed of the eight main species of P450 families of *Inonotus obliquus*

According to domain and pfam prediction, 135 P450 genes were screened in the *I. obliquus* genome (Table S15). Based on family cluster analysis of 135 CYPs, it was found that 107 genes could be clustered and divided into 19 families. Especially 8 families contained CYP620 (24), CYP512 (14), CYP5150 (10), CYP5154 (9), CYP5141 (5), CYP5144 (11), CYP5037 (8), and CYP5035 (7) families (Fig. 8). These cytochrome P450 subfamilies could be closely related to the formation of secondary metabolites in *I. obliquus*. There are major bioactive compounds in *I. obliquus*, including inotodiol, betulin, and betulinic acid. These compounds represent two different types of triterpenes. The synthesis of lanosterol and lupeol are respectively catalyzed by lanosterol synthase and lupeol synthase enzymes, with 2,3-oxidosqualene as a precursor. Lanosterol produces inotodiol via the action of cytochrome P450 hydroxylation, and lupeol produces betulin and betulinic acid through the combined action of cytochrome P450 oxidase and reductase; however, cytochrome P450 and lupeol synthase have not been reported in *I. obliquus* and other fungi*.* We only choose the sequences in plants based on the synthesis of the same or similar substances according to the reference. Specifically, Yang et al. reported that CYP89S1, CYP97B62, and CYP86A182 have C-28 oxidation functions and catalyze the conversion of lupeol to betulinic acid in birch [31]; further, CYP90B and CYP724B have C-22 hydroxylation functions and catalyze the formation of steroids in plants such as *Arabidopsis thaliana* and *Solanum tuberosum* [32–35]. Owing to the structural similarity of triterpenes and steroids, inotodiol synthesis includes lanosterol C22 hydroxylation. Therefore, according to BLASTP screening, similar related P450 sequences in the genome (Table S14) were hypothesized, comparing sequences of all P450s, which provides a foundation for further experimental verification in a later stage. Based on different gene families from phylogenetic tree analysis, fungal CYPs showed highly conserved characteristic motifs but very low overall sequence similarities [36]. Betulinic acid biosynthesis related to P450, g5553 and g3231 were determined to belong to the same family, CYP63, and g7106, g6587, g8846 were respectively CYP5032, CYP5148, CYP5037 families. Inotodiol biosynthesis is related to P450, mainly distributed the CYP51 and CYP512 subfamilies (Table S14).

## Discussion

Herein is described that the assembly and annotation of *I. obliquus* genome. Using the latest third-generation sequencing technology, the Oxford Nanopore PromethION sequencing platform, the genome sequence of *I. obliquus* was analyzed, utilizing NECAT software to perform genome error correction and splicing and finally obtaining initial joint results. Racon (version:1.4.11) software was used twice based on joint results for error correction. Finally, Pilon (version:1.23) software was used twice for error correction after purging the haplotigs to obtain the final assembled results. The average size of most mushroom genomes is approximately 40 Mb, and the size of the assembled *I. obliquus* genome (38.18 Mb) conformed to expectations based on the closest *Sanghuangporus* genome (34.5 Mb). We found 380 carbohydrate-related genes in the genome of this fungus. From the perspective of the number of degrading enzymes, *I. obliquus*, as a white-rot fungus, also has a strong ability to degrade lignocellulose. Although the polarity of many species from Agaricales and Polyporus has been analyzed [20], sufficient reports on the polarity of Hymenochaetales are currently lacking. It has been reported that *F. mediterranea* is biopolar [37]. The polarity of *I. obliquus* is based on the distribution of its reproduction-related genes on the contigs, and we infer that it might be tetrapolar.

In this study, we uncovered and annotated important genes related to its secondary metabolism. polysaccharides comprise one of the major categories of pharmacologically active compounds in macrofungi. Some key genes for polysaccharide synthesis have been reported in other medicinal mushrooms. For example, the overexpression of phosphoglucomutase can increase the polysaccharide content in *G. lucidum* [38]. In *C. militaris*, co-expressed phosphoglucomutase and UDP-glucose 6-dehydrogenase can improve the whole content of intracellular and extracellular polysaccharides, increasing polysaccharide content by 78.13% compared with that of the wild-type strain [39]. Exogenous siRNAs were also previously applied to target β-1,3-glucan synthase, negatively affecting the growth of the fungus *Macrophomina phaseolina*. Fungal cell walls are composed of chitin and glucan; therefore, polysaccharide synthesis is strongly correlated with regular hyphal growth [40]. The metabolism of polysaccharides found in *I. obliquus* is similar to that of other medicinal fungi. In recent years, there has been increasing research on PKSs in basidiomycetes owing to attention being paid to the biosynthesis of polyketide active compounds in basidiomycetes. For example, PKS1 from *C. cinerea* was heterologously expressed in *Saccharomyces cerevisiae*, where it catalyzed the formation of orsellinic acid [23]. PKS63787 is responsible for the biosynthesis of orsellinic acid in *A. cinnamomea* [41]. A PKS was also found in *I. obliquus*, with 39% and 29% identical amino acids compared to PKS63787 and PKS1, which could be involved in orsellinic acid biosynthesis, but no such compound has been reported among the secondary metabolites of *I. obliquus*. Kwang reported a novel tripeptide with a molecular mass of 365 Da and a sequence of Trp-Gly-Cys [42]. There are four NRPSs in *I. obliquus* that might be responsible for the production of this compound, but the function of NPRSs of basidiomycetes has not been reported to date, and this needs to be explored.

In this article, 20 total genes related to sesquiterpene synthase were discovered and the surrounding genes were annotated. However, according to relevant references, only eight sesquiterpenoids have been found in *I. obliquus* [43, 44]. The number of sesquiterpenes reported to date is much smaller than the original number of encoding genes. We speculate that many genes might be in the silent stage. The discovery of these genes will help to study the biosynthetic pathways of sesquiterpenoid secondary metabolites in *I. obliquus*. Transcriptome research of *I. obliquus* revealed three different types of triterpene synthases [17], but we found only one lanostane-type triterpene synthase (lanostane synthase) in the genome. The key enzyme required for the synthesis of lupane triterpenoids by *I. obliquus* has not been found yet. For example, lupane triterpenoid betulinic acid is mostly found in plants, such as birch [31] and mulberry [45]. However, it is rarely reported in fungi, except for *S. baumii* [46], *Trametes versicolor* [47], and *I. obliquus*. We need to identify genes related to its biosynthesis to establish a consensus.

P450 plays an important role in the biosynthesis of secondary metabolites in mushrooms and is involved in triterpene synthesis hydroxylation, carbonylation, carboxylation, and ketonation. Regarding P450, in the medicinal and edible mushroom *G. lucidum*, 219 CYP genes (197 functional genes and 22 pseudogenes) were found, divided into 42 families [12]. *A. cinnamomea* harbors 119 CYP genes [13], *Hypsizygus marmoreus* has 132 CYP genes [48], and *H. erinaceus* contains 137 CYP genes [49]. Our study found a total 135 P450 genes in *I. obliquus* and eight different families of P450s are displayed. In *G. lucidum*, CYP512 family proteins might be involved in triterpenoid biosynthesis [12]. CYP5150A2 from the white-rot basidiomycete *P. chrysosporium* is capable of hydroxylating 4-propylbenzoic acid with NADPH-dependent cytochrome P450 oxidoreductase as a single redox partner [50]. In *I. obliquus*, we found 12 genes from the CYP512 family, seven genes belonging to the CYP5035 family and 10 genes from the CYP5150 family, which might be involved in the biosynthesis of terpenoids. Functional screening showed that CYP5035 assists in the fungal detoxification mechanism in Polyporales [51]. We analyzed candidate P450 proteins related to betulinic acid and inotodiol synthesis. Inotodiol biosynthesis involved two P450s belong to the CYP51 family. Zhang et al. reported that CYP51 belongs to the CYP superfamily and is a crucial step in the synthesis of ergosterol, which is a fungal-specific sterol. CYP51 has strong specificity and only catalyzes the demethylation of a very narrow range of substrates, including lanosterol [52]. So, different types of P450 are essential for secondary metabolites biosynthesis in *I. obliquus.*

## Conclusion

In this study, we presented the first genome analysis of an important medical mushroom, *I. obliquus*. For the de novo sequenced and annotated genome, assembled using the Oxford Nanopore PromethION sequencing platform, detailed functional annotations were made for the genome of *I. obliquus* using major databases. The information on the *I. obliquus* genome could provide a clear genetic background for the study of secondary metabolism and its medicinal applications. We analyzed the secondary metabolite biosynthesis genes in the *I. obliquus* genome, such as key genes related to polysaccharides, melanin and terpenoid. Additionally, we identified some candidate P450 proteins related to betulinic acid and inotodiol biosynthesis.

## Methods

### Collection of strains and culture conditions

The *I. obliquus* strain was obtained from the Microbiology Laboratory, College of Life Science, Northeast Forestry University. The fruit body was collected from the Greater Khingan Mountains area and named CT5, which was identified based on internal transcribed spacer sequences (ITS1 and ITS4) after tissue separation. The strain was cultured on potato-dextrose broth at 30 °C for 5 days. The *I. obliquus* genomic DNA was extracted from mycelia using the Tiangen plant DNA kit DP350, according to the manufacturer’s instructions.

### Genome sequencing and assembly

After the library was built, an effective concentration and volume of the DNA library was added to the flow cell, and was transferred to the Oxford Nanopore PromethION sequencer with Illumina NovaSeq [53] for real-time single-molecule sequencing (NCBI SRA database accession number SRR15674625). The genome size of *I. obliquus* was estimated by the k-mer method using sequencing data from the DNA library. The Oxford Nanopore PromethION sequenator was supported by the software Guppy to automatically distinguish between Pass and Fail data. Illumina NovaSeq filtration was used with fastp software (https://github.com/OpenGene/fastp). The Oxford Nanopore PromethION filter criteria were as follows: 1) remove sequences for which the average mass value is less than or equal to 7. Illumina NovaSeq filtration standard: 1) remove reads with an N base content exceeding 5%; 2) remove reads of low quality (mass value less than or equal to 5) with a 50% base number; 3) remove reads contaminated by Adapter; 4) remove the repeated sequences caused by PCR amplification. NECAT software[54] was used to perform genome error correction and splicing was performed to obtain the initial splicing result; then, Racon (version: 1.4.11) software [55] was used to perform two rounds of error correction on the splicing result based on the third-generation sequencing data, and finally, two rounds of Pilon were performed (version: 1.23). Error correction was performed [56], and after removing heterozygosity, the final assembly result was obtained. BUSCO software (version: 4.1.4) was used to evaluate the integrity of the predicted genes based on the fungal database (fungi_odb10) (v.4.0.6) [57].

### Gene prediction and annotation

Gene prediction was performed mainly using BRAKER software (version: 2.1.4); first, GeneMark-EX was used to train the model, and then, AUGUSTUS was called for prediction [58]. INFERNAL (Version: 1.1.2) was used to predict and classify ncRNA based on the Rfam database. Repetitive sequences can be divided into scattered repeats and tandem repeats. Scattered repeating sequences, also known as transposon elements, include four types, LTR, LINE, SINE, and DNA transposons. According to the number of repetitions, they can be divided into highly repetitive sequences, moderately repetitive sequences, and low repetitive sequences. RepeatModeler software (Version: 1.0.4) was used to build its own repeat library, and RepeatMasker (version: 4.0.5) was used to annotate the repeated sequence of the genome after merging the repbase library.

Gene function annotation referred to the annotation of gene functions and metabolic pathways based on existing databases, including predictions of information such as motifs, structural domains, protein functions, and metabolic pathways. Gene annotations were refined using the following databases: Nr, Pfam [59], COG [60], Uniprot [61], KEGG [62], GO [63], Pathway, Refseq [64], and Interproscan [65]. Gene function annotation was performed using two main methods as follows: (1) sequence similarity search: the protein sequence of genome was aligned with the existing protein databases Uniprot, Refseq, NR, and KEGG (metabolic pathway database) for diamond blastp (version: 2.9.0; parameter: –evalue 1e-5) to obtain the functional information of sequences, as well as information on the metabolic pathways in which the protein is probably. KEGG annotations were associated with KEGG ORTHOLOGY and PATHWAY using KOBAS (version: 3.0). The Uniprot database records the correspondence between each protein family and the functional node in GO and the biological function performed based on the protein sequence. Based on the association between the databases (Uniprot/Swiss-Port), we obtained the annotation information of the eggNOG database, selected the COG annotation results, and performed COG classification statistical analysis and drawing. (2) Motif similarity search: we used hmmscan(version: 3.1; parameter: e-value 0.01) to predict structural domains to obtain conserved sequences, motifs, and domains of the protein. The Pfam database is a large collection of protein families, depending on multiple sequence alignments and thee Hidden Markov Model. The protein sequence of the genome was aligned with second databases, including InterPro subdata CDD-3.16, Coils-2.2.1, Gene3D-4.2.0, Hamap-2018_03, MobiDBLite-2.0, Pfam-32.0, PIRSF-3.02, PRINTS-42.0, ProDom-2006.1, ProSitePatterns-2018_02, ProSiteProfiles-2018_02, SFLD-4, SMART-7.1, SUPERFAMILY-1.75, and TIGRFAM-15.0 based on InterProScan (version: 5.33–72.0) to obtain conserved sequences, motifs, and domains of the protein.

### Phylogenetic location

Together with *I. obliquus* and other 19 species (*Gloeophyllum trabeum*, *Fomitopsis pinicola*, *Lentinula edodes*, *Pleurotus ostreatus*, *S. baumii*, *Fomitiporia mediterranea*, *W. cocos*, *Dichomitus squalens*, *Coprinopsis cinerea*, *Schizophyllum commune*, *Phanerochaete chrysosporium*, *Agaricus bisporus*, *Ustilago maydis*, *Stereum hirsutum*, *Trametes versicolor*, *Laccaria bicolor*, *Aspergillus oryzae*, *A. nidulans*, and *A. niger*), homologous gene identification and phylogenetic analysis were performed. Single-copy homologous genes were identified using OrthoFinder version: 2.3.12, with the default inflation value (1.5) [66]. STAG 1.0 was used to build a phylogenetic tree [67], and then, MCMCtree (is a program from paml 4.9j) was utilized to predict divergence time [68]. Two groups of recent ancestor divergence times were queried as calibrated points in timetree.org [69] (http://www.timetree.org/) (*A. niger* vs. *A. bisporus* 626–806 MYA and *A. bisporus* vs. *U. maydis* 415–482 MYA).

### Identification of *matA* and *matB* genes

Using tetrapolarity *S. commune MAT-A* genes as a reference with pfam domain to predict and identify conserved domains [70], we identified *MAT-A* genes in the genome. The mitochondrial intermediate peptidase gene (*mip*) was identified in the same manner. *MAT-B* genes include pheromone and pheromone precursors. The sequence length of the pheromone precursor was too short to align it for prediction. We used an annotation file to find the *MAT-A*-and *MAT-B*-specific locations.

### CAZy and CYP family in *I. obliquus*

Carbohydrates play an important role in many biological processes. A large amount of meaningful biological information can be obtained by studying carbohydrate-related enzymes. CAZy data focus on analyzing the genome, structure, and biochemical information of carbohydrate enzymes (Table S16). HMMER (version: 3.2.1, filter parameter E-value < 1e^−18^; coverage > 0.35) [71] was used to annotate protein sequences based on the CAZy database (http://bcb.unl.edu/dbCAN2/) [72].

Cytochrome P450 is a large family of proteins, with heme as a prosthetic group. They can catalyze the oxidation reactions of many types of substrates, and they participate in the metabolism of endogenous and exogenous substances, including drugs and environmental compounds. Diamond blastp (version > 2.9.0; parameter: –evalue 1e^−5^) was used to annotate the target protein sequence based on the Fungal cytochrome P450 database. The reference CYP sequences were downloaded from the web (http://p450.riceblast.snu.ac.kr/index.php?a=view) [73].

### Prediction of gene clusters involved in secondary metabolites

Secondary metabolite gene clusters were predicted using 2ndFind (http://biosyn.nih.go.jp/2ndFind/) a web-based analytical tool, and antiSMASH 6.0 platforms (http://antismash.secondarymetabolites.org/) [74], a web-based analysis platform. AntiSMASH currently offers a broad collection of tools and databases for automated genome mining and comparative genomics for a wide variety of different classes of secondary metabolites. The default parameter settings were used. To verify the predicted results, the obtained gene clusters were manually checked. Blastp analysis and gene annotation were performed using the NCBI genome portal software platform. We searched all hypothetical gene models in the database using blastp and tblastn algorithms.

### Bioinformatics and phylogenetic analyses of PKSs, STSs, and P450s.

32 homologous PKS sequences of different fungal species that have been functionally verified to be involved in the production of orsellinic acid or melanin were retrieved from the National Center for Biotechnology Information and JGI database. For phylogenetic analysis, the KS domain sequences from functional or putative PKSs involved in the biosynthesis of melanins were aligned using the program Clustal X (Version 2.0), and a maximum-likelihood tree was generated using MEGA (Version 10.0) software. In order to classify 20 STSs in *I. obliquus*, we selected 32 sesquiterpenes from *O. olearius*, *S. hirsutum*, and *C. cinereu*s as reference, and 1000 bootstraps were used to establish a compared 52 sequences maximum-likelihood tree using MEGA. Three similar species (*C. cinerea*, *A. bisporus*, and *P. ostreatus*) were selected from the fungal P450 database, the P450 gene sequences were selected as references for comparisons, and the P450s in *I. obliquus* were clustered. Phylogenetic tree analysis was performed on 88 P450s with a large number and clear classification in the same manner.

## Supplementary Information

**Additional file 1.** 

**Additional file 2.** 

## Data Availability

The *Inonotus obliquus* genomic data have been deposited under accession JAIHLT000000000 in GenBank. The version described in this paper is version JAIHLT010000000. The genome raw sequencing data and the reported assembly are associated with NCBI BioProject: PRJNA754990 and BioSample: SAMN20834359 within GenBank.

## Abbreviations

AAs: Auxiliary activities
CAZymes: Carbohydrate-active enzymes
CE: Carbohydrate esterase
COG: Clusters of Orthologous Groups
CYP: Cytochrome P450 monooxygenase
DHN: 1,8-Dihydroxynaphthalene
FPP: Farnesyl diphosphate
GH: Glycoside hydrolase
GO: Gene ontology
GT: Glycosyltransferase
HD: Homeodomain
KEGG: Kyoto Encyclopedia of Genes and Genomes
Mya: Million years ago
PL: Polysaccharide lyase
STS: Sesquiterpene synthases
