# Supplementary material for: Genome sequencing of Inonotus obliquus reveals insights into candidate genes involved in secondary metabolite biosynthesis
Source: BMC Genomics. 2022 Apr 20;23:314. doi: 10.1186/s12864-022-08511-x (PMC9020118; doi:10.1186/s12864-022-08511-x)
Supplement: Supplementary file 1 — Additional file 1. [file 12864_2022_8511_MOESM1_ESM.docx]

***Supplementary Tables***

**Genome sequencing of *Inonotus obliquus* reveals insights into candidate genes involved in secondary metabolite biosynthesis**

Yingce Duan^1^, Haiyan Han^1^, Jianzhao Qi^2^, Jin-ming Gao^2^, Zhichao Xu^1^, Pengchao Wang^1^, Jie Zhang^1^, Chengwei Liu^1*^

Supplementary tables (16 total)

**Table S1** **Statistics of Oxford Nanopore PromethION** **sequencing data volume of *Inonotus obliquus* genome**

| **Rank** | **Flag** | **TotalBase** | **TotalReads** | **MaxLen** | **AvgLen** | **N50** | **L50** | **N90** | **L90** | **meanQ** |
| --- | --- | --- | --- | --- | --- | --- | --- | --- | --- | --- |
| >0 | all | 7,730,063,186 | 1,112,054 | 211,032 | 6,951.15 | 15,050 | 157,280 | 2,975 | 613,156 | 10.86 |
| >0 | pass | 7,437,204,734 | 1,066,574 | 108,439 | 6,972.98 | 15,062 | 151,282 | 2,984 | 589,043 | 11.06 |
| >0 | fail | 292,858,452 | 45,480 | 211,032 | 6,439.27 | 14,629 | 6,002 | 2,798 | 24,148 | 6.27 |
| >5000 | all | 6,238,302,755 | 426,397 | 211,032 | 14,630.26 | 18,688 | 112,826 | 7,247 | 322,658 | 10.83 |
| >5000 | pass | 6,005,082,906 | 410,397 | 108,439 | 14,632.37 | 18,691 | 108,632 | 7,249 | 310,553 | 11.00 |
| >5000 | fail | 233,219,849 | 16,000 | 211,032 | 14,576.24 | 18,628 | 4,195 | 7,195 | 12,105 | 6.29 |
| >10000 | all | 4,964,828,792 | 246,481 | 211,032 | 20,142.84 | 22,038 | 81,447 | 12,243 | 201,659 | 10.77 |
| >10000 | pass | 4,779,941,336 | 237,317 | 108,439 | 20,141.58 | 22,036 | 78,445 | 12,242 | 194,166 | 10.95 |
| >10000 | fail | 184,887,456 | 9,164 | 211,032 | 20,175.4 | 22,067 | 3,002 | 12,256 | 7,493 | 6.29 |
| >50000 | all | 114,011,287 | 2,005 | 211,032 | 56,863.48 | 55,020 | 913 | 50,761 | 1,779 | 10.26 |
| >50000 | pass | 107,450,640 | 1,900 | 108,439 | 56,552.96 | 54,956 | 870 | 50,758 | 1,687 | 10.49 |
| >50000 | fail | 6,560,647 | 105 | 211,032 | 62,482.35 | 57,029 | 43 | 50,939 | 92 | 6.28 |
| >100000 | all | 1,081,026 | 8 | 211,032 | 135,128.25 | 124,209 | 3 | 102,763 | 7 | 6.29 |
| >100000 | pass | 312,455 | 3 | 108,439 | 104,151.66 | 102,763 | 2 | 101,253 | 3 | 9.34 |
| >100000 | fail | 768,571 | 5 | 211,032 | 153,714.2 | 208,802 | 2 | 106,119 | 5 | 4.45 |

**Rank** is the gradient of data length, **>0** is that all data; **Flag** is data type, **all** is all sequencing data, **pass** is effective sequencing data, **fail** is filtration data; **TotalBase** is the number of base; **TotalReads** is the number of reads; **MaxLen** is maximum length of data; **AvgLen** is average length of data; **N50** is N50 of data, all reads are summed in order from long to short, and when additive length up to half of all reads total length, the last read length added is N50; **L50** is L50 of data, all reads are accumulated in turn ranking in order from long to short, when additive length up to half of all reads total length, the number of sequences is L50; **N90** is N90 of data, algorithm the same as N50; **L90** is L90 of data, algorithm the same asL50; **meanQ** is mean quality value.

**Table S2 Statistics of Illumina NovaSeq sequencing data volume information of *Inonotus obliquus* genome**

| **Sample_name** | **Total_reads** | **Total_bases** | **GC_content** | **Q20** | **Q30** |
| --- | --- | --- | --- | --- | --- |
| rawdata | 37,219,614 | 5,582,942,100 | 47.44% | 98.28% | 94.86% |
| cleandata | 37,218,262 | 5,582,739,300 | 47.44% | 98.28% | 94.86% |

**Sample name** is data type; **Total_reads** is sequencing reads number; **Total_bases** is total sequencing base number; **GC_Content** is G/C base number as a percentage of total base number; **Q20、Q30** respectively are Phred value greater than 20、30 base as a percentage of total base.

**Table S3** **Statistics of Illumina NovaSeq sequencing data mapping of *Inonotus obliquus* genome**

| **Type** | **Number** |
| --- | --- |
| map_rate | 99.21% |
| Average_depth | 143.47 |
| Coverage | 99.57% |

**map_rate** is Illumina NovaSeq sequencing data mapping rates; **Average_depth** is average coverage depth; **Coverage** is coverage rate.

**Table S4** **Statistics of BUSCO evaluation of *Inonotus obliquus* genome**

| **Item** | **Number** | **Percent(%)** |
| --- | --- | --- |
| Complete BUSCOs (C) | 666 | 87.9 |
| Complete and single-copy BUSCOs (S) | 642 | 84.7 |
| Complete and duplicated BUSCOs (D) | 24 | 3.2 |
| Fragmented BUSCOs (F) | 16 | 2.1 |
| Missing BUSCOs (M) | 76 | 10.0 |
| Total BUSCO groups searched | 758 | 100.0 |

**Table S5** **Annotation Statistics of *Inonotus obliquus* coding gene**

| **Type** | **Number** |
| --- | --- |
| Total number of gene | 12,525 |
| Average of mRNA length | 1,742.89 |
| Average of cds length | 1,296.52 |
| Average of exon number | 7.19 |
| Average of exon length | 180.37 |
| Average of intron length | 72.13 |
| Total number of exon | 90,032 |
| Total number of intron | 77,507 |
| Total intron length | 5,590,447 |

**Table S6 Statistics of non-coding RNA annotation results in *Inonotus obliquus* genome**

| **Class** | **number** | **totalLen(bp)** | **meanLen(bp)** |
| --- | --- | --- | --- |
| rRNA | 78 | 138,037 | 1,769 |
| sRNA | 1 | 346 | 346 |
| snRNA | 14 | 2,036 | 145 |
| tRNA | 88 | 7,175 | 81 |

**totalLen** and **meanLen** are the total length and mean length

**Table S7 Statistics of *Inonotus obliquus* repetitive sequence annotation results**

| **Item** | **Subfamily** | **Number** | **Length(bp)** | **Coverage** |
| --- | --- | --- | --- | --- |
| SINE | / | 4 | 470 | 0.00% |
| LINE | / | 566 | 563,022 | 1.47% |
| LTR | / | 1,286 | 1,488,734 | 3.90% |
| LTR | Gypsy | 931 | 1,226,998 | 3.21% |
| LTR | Copia | 268 | 256,041 | 0.67% |
| DNA | / | 732 | 345,311 | 0.90% |
| Satellite | / | 11 | 888 | 0.00% |
| Simple_repeat | / | 5,401 | 224,065 | 0.59% |
| Low_complexity | / | 865 | 43,383 | 0.11% |
| Other | / | 44 | 7,940 | 0.02% |
| Unknown | / | 8,975 | 5,606,972 | 14.69% |
| Total | / | 17,884 | 8,227,984 | 21.55% |

**SINE** is short scattered element; **LINE** is Long scattered element; **LTR** is long terminal repetition, mainly include two types, Gypsy and Copia; **DNA** is transposons; **Satellite** is satellite repetitive sequence; **Low_complexity** is Low_complexity repetition; **Other** is other types repetition; **Unknown** is unknown repetitive sequence; **Total** is total repetitive sequence.

| **Species** | **Source** |
| --- | --- |
| *Inonotus obliquus* | in the study |
| *Aspergillus nidulans* | https://genome.jgi.doe.gov/portal/pages/dynamicOrganismDownload.jsf?organism=Aspnid1 |
| *Aspergillus niger* | https://genome.jgi.doe.gov/portal/pages/dynamicOrganismDownload.jsf?organism=Aspni_NRRL3_1 |
| *Aspergillus oryzae* | https://genome.jgi.doe.gov/portal/pages/dynamicOrganismDownload.jsf?organism=Aspor1 |
| *Coprinopsis cinerea* | https://genome.jgi.doe.gov/portal/pages/dynamicOrganismDownload.jsf?organism=Copci1 |
| *agaricus bisporus* | https://genome.jgi.doe.gov/portal/Agabi_varbisH97_2/Agabi_varbisH97_2.download.ftp.html |
| *Sanghuangporus baumii* | https://www.ncbi.nlm.nih.gov/genome/?term=Sanghuangporus+baumii |
| *Trametes versicolor* | https://www.ncbi.nlm.nih.gov/genome/?term=Trametes+versicolor |
| *Laccaria bicolor* | https://genome.jgi.doe.gov/portal/pages/dynamicOrganismDownload.jsf?organism=LacbiH82_1 |
| *Lentinula edodes* | https://genome.jgi.doe.gov/portal/pages/dynamicOrganismDownload.jsf?organism=Lentinedodes1 |
| *Pleurotus ostreatus* | https://mycocosm.jgi.doe.gov/PleosPC15_2/PleosPC15_2.home.html |
| *Dichomitus squalens* | https://genome.jgi.doe.gov/portal/pages/dynamicOrganismDownload.jsf?organism=Dicsqu463_1 |
| *phanerochaete chrysosporium* | https://mycocosm.jgi.doe.gov/Phchr2/Phchr2.home.html |
| *Schizophyllum commune* | https://genome.jgi.doe.gov/portal/pages/dynamicOrganismDownload.jsf?organism=Schco3 |
| *Fomitiporia mediterranea* | https://genome.jgi.doe.gov/portal/pages/dynamicOrganismDownload.jsf?organism=Fomme1 |
| *Wolfiporia cocos* | https://genome.jgi.doe.gov/portal/pages/dynamicOrganismDownload.jsf?organism=Wolco1 |
| *Fomitopsis pinicola* | https://genome.jgi.doe.gov/portal/pages/dynamicOrganismDownload.jsf?organism=Fompi3 |
| *Gloeophyllum trabeum* | https://www.ncbi.nlm.nih.gov/genome/?term=Gloeophyllum+trabeum |
| *Ustilago maydis* | http://www.ncbi.nlm.nih.gov/genome/?term=Ustilago+maydis |
| *Stereum hirsutum* | https://www.ncbi.nlm.nih.gov/genome/?term=Stereum+hirsutum |

**Table S8** **Statistics from 20 fungal species phylogenetic tree sources**

**Table S9 Candidate genes for mating type** **in *Inonotus obliquus* genome**

| **Top Annotation** | **Gene ID** | **Species** | **E -Value** | **Accession Number** |
| --- | --- | --- | --- | --- |
| mitochondrial intermediate peptidase | g5642  g5643 | *Schizophyllum commune* | 0.0  1e^-064^ | EFJ03821.1 |
| homeodomain protein | g5644  g5645  g5646 | *Pleurotus djamor*  *Pleurotus djamor*  *Schizophyllum commune* | 4e^-023^  6e^-019^  8e^-010^ | AAS46746.1  AAS46746.1  AAB41340.1 |
| FMN-linked oxidoreductase | g5647 | *Sparassis crispa* | 9e^-007^ | XP_027614887.1 |
| glycosyltransferase family 8 protein | g5659 | *Sparassis crispa* | 7e^-004^ | GBE79089.1 |
| beta-flanking gene | g5660 | *Lentinula edodes* | 4e^-052^ | AEN14452.1 |
| STE3-like pheromone receptor  （STE） | g8676  g8438  g8458 | *Pleurotus ostreatus* | 5e^-053^  6e^-033^  2e^-027^ | AKQ62722.1 |

**Table S10 Distribution of polysaccharide genes in *Inonotus obliquus* genome**

| **Top Annotation** | **Gene ID** | **Species** | **E -Value** | **Accession Number** |
| --- | --- | --- | --- | --- |
| Glucokinase | g4592  g905 | *Saccharomyces cerevisiae* | 2e^-73^  9e^-70^ | NP_009890.1  NP_009890.1 |
| Phosphoglucomutase/ Glucose phosphomutase | g1812 | *Laccaria bicolor* | 0 | EDR14589.1 |
| UTP-glucose-1-phosphate uridylyltransferase | g2662  g2663 | *Cordyceps militaris* | 3e^-38^  9e^-10^ | ATY59765.1  ATY59765.1 |
| 1,3-beta-glucan synthase | g934  g445 | *Pleurotus eryngii* | 0  0 | KAF9500877.1  KAF9500877.1 |
| Beta-glucan synthesis-associated protein | g4208  g9944  g4340  g5843  g8817 | *Sparassis crispa* | 0  0  e^-143^  e^-137^  7e^-25^ | GBE78044.1  GBE78044.1  GBE78044.1  GBE78044.1  GBE78044.1 |
| Phosphomannose isomerase/Mannose-6-phosphate isomerase | g9920  g9214 | *Aspergillus nidulans* | 5e^-60^  5e^-33^ | AAA33319.1  AAA33319.1 |
| Glucose-6-phosphate isomerase | g788 | *Aspergillus fumigatus* | 0 | KEY79565.1 |
| Fructose-1,6-bisphosphatase | g311 | *Saccharomyces cerevisiae* | 9e^-97^ | P09201.2 |
| UDP-glucose-6-dehydrogenase | g1235 | *Trichoderma guizhouense* | e^-123^ | OPB40409.1 |
| UDP-glucose 4-epimerase | g5562 | *Monascus purpureus* | 2e^-72^ | TQB76668.1 |

**Table S11 Basidiomycete and Ascomycetes species whose sequenced genomes were investigated by antismash in this study.**

| **Species** | **PKS** | **PKS-NRPS** | **NRPS** | **Terpene** |
| --- | --- | --- | --- | --- |
| *Inonotus obliquus* | **1** | **2** | **4** | **12** |
| *Sanghuangporus baumii* | **1** | **2** | **3** | **12** |
| *Fomitiporia mediterranea* | **2** | **2** | **5** | **19** |
| *Coprinopsis cinerea* | **2** | **--** | **3** | **9** |
| *Schizophyllum commune* | **1** | **--** | **9** | **5** |
| *Gloeophyllum trabeum* | **6** | **3** | **12** | **12** |
| *Dichomitus squalens* | **3** | **--** | **13** | **14** |
| *Phanerochaete chrysosporium* | **2** | **2** | **16** | **13** |
| *Laccaria bicolor* | **1** | **--** | **4** | **8** |
| *Agaricus bisporus* | **1** | **1** | **6** | **10** |
| *Pleurotus ostreatus* | **1** | **--** | **8** | **17** |
| *Stereum hirsutum* | **3** | **2** | **19** | **18** |
| *Trametes versicolor* | **2** | **--** | **10** | **15** |
| *Wolfiporia cocos* | **6** | **1** | **7** | **19** |
| *Lentinula edodes* | **4** | **--** | **10** | **8** |
| *Fomitopsis pinicola* | **4** | **4** | **4** | **19** |
| *Ustilago maydis* | **1** | **--** | **10** | **2** |
| *Aspergillus nidulans* | **17** | **4** | **19** | **12** |
| *Aspergillus niger* | **26** | **15** | **27** | **18** |
| *Aspergillus oryzae* | **26** | **5** | **25** | **10** |

**Table S12 The analyzed PKSs in phylogenetic tree.**

| Number | Species | Protein | Accession number | Chemical product |
| --- | --- | --- | --- | --- |
| 1 | *Antrodia cinnamomea* | PKS63787 | AST08390.1 | Orsellinic acid |
| 2 | *Coprinopsis cinerea* | PKS1 | XP_001835415.2 | Orsellinic acid |
| 3 | *Ustilago maydis* | PKS3 | XP_011390610.1 | Orsellinic acid |
| 4 | *Laetiporus sulphureus* | LpaA | QNJ99675.1 | laetiporic acid |
| 5 | *Inonotus obliquus* | g7818 | OM777187 | In the study |
| 6 | *Aspergillus oryzae* | PKSA | BAE71314.1 | Norsolorinic acid |
| 7 | *Aspergillus fumigatus* | PksP | Q4WZA8.1 | DHN-melanin |
| 8 | *Agaricus bisporus* | PKS1 | XP_006460038.1 | Unknown |
| 9 | *Trametes versicolor* | PKS1 | XP_008041566.1 | Unknown |
| 10 | *Schizophyllum commune* | PKS1 | XP_003038401.1 | Unknown |
| 11 | *Trametes chinabarina* | PKS1 | CDO73915.1 | Unknown |
| 12 | *Gymnopus luxurians* | PKS1 | KIK51473.1 | Unknown |
| 13 | *Grifola frondosa* | PKS1 | BAO20284.1 | Unknown |
| 14 | *Fusarium graminearum* | PKS14 | EYB32182.1 | Orcinol |
| 15 | *Fusarium graminearum* | PKS13 | EYB31054.1 | Zearalenone |
| 16 | *Fusarium graminearum* | PKS12 | EYB30989.1 | Aurofusarin |
| 17 | *Fusarium graminearum* | PKS3 | EYB26831.1 | Fusarubin |
| 18 | *Amanita muscaria* | PKS1 | KIL57002.1 | Unknown |
| 19 | *Fusarium fujikuroi* | PKS3 | CAC88775.1 | Fusarubin |
| 20 | *Fomitopsis pinicola* | PKS1 | EPS94471.1 | Unknown |
| 21 | *Fibroporia radiculosa* | PKS1 | XP_012179384.1 | Unknown |
| 22 | *Aspergillus nidulans* | PkgA | CBF79143.1 | Alternariol |
| 23 | *Aspergillus nidulans* | PkfA | CBF83139.1 | Aspernidine |
| 24 | *Aspergillus nidulans* | PkdA | CBF89312.1 | Orsellinaldehyde |
| 25 | *Aspergillus nidulans* | AfoE | Q5BEJ6.1 | Asperfuranone |
| 26 | *Aspergillus nidulans* | AptA | Q5B0D0.1 | Asperthecin |
| 27 | *Aspergillus nidulans* | AusA | Q5ATJ7.1 | Austinol |
| 28 | *Aspergillus nidulans* | MdpG | CBF90097.1 | Emodin |
| 29 | *Botrytis cinerea* | Bcpks13 | XP_001547095.2 | DHN-melanin |
| 30 | *Botrytis cinerea* | Bcpks12 | XP_024547375.1 | DHN-melanin |
| 31 | *Metarhizium robertsii* | MrPKS1 | XP_007823934.2 | Quinone pigments |
| 32 | *Talaromyces marneffei* | PKS4 | ADH01664.1 | Quinone pigments |
| 33 | *Neurospora crassa* | NCU03584 | EAA31350.3 | DHN-melanin |
| 34 | *Stereum hirsutum* | PKS1 | XP_007307184.1 | Unknown |
| 35 | *Alternaria alternata* | Alm | AEH76763 | melanin |

**Table S13 Terpenoid biosynthesis related enzymes in *Inonotus obliquus* genome.**

| **Top Annotation** | **Gene ID** | **Species** | **E -Value** | **Accession Number** |
| --- | --- | --- | --- | --- |
| Acetyl-CoA acetyltransferase | g4782  g5768  g2547  g5993 | *Sparassis crispa* | e^-171^  1e^-054^  9e^-052^  5e^-048^ | XP_027610232.1  XP_027610232.1  XP_027610232.1  XP_027610232.1 |
| 3-hydroxy-3-methyl glutaryl-CoA synthase | g1923 | *Neurospora crassa* | 3e^-062^ | BAJ83614.1 |
| 3-hydroxy-3-methyl glutaryl-CoA reductase | g3754 | *Neurospora crassa* | e^-153^ | BAJ83615.1 |
| mevalonate kinase | g9045  g7463 | *Cordyceps javanica*  *Cordyceps javanica* | 9e^-068^  9e^-068^ | TQV90862.1  TQV90862.1 |
| phosphomevalonate kinase | g11342 | *Saccharomyces cerevisiae* | 5e^-050^ | NP_013947.1 |
| mevalonate pyrophosphate decarboxylase | g11254 | *Sanghuangporus baumii* | e^-174^ | QDM54308.1 |
| isopentenyl diphosphate isomerase | g6202 | *Ganoderma lucidum* | e^-120^ | AGL94943.1 |
| diterpene synthase/sesterterpene synthases/ Geranylgeranyl diphosphate synthase | g7179 | *Penicillium chrysogenum* | 1e^-054^ | BBD06033.1 |
| farnesyl diphosphate synthase | g8793  g8794 | *Ganoderma lucidum* | 3e^-055^  1e^-039^ | ACB37021.1  ACB37021.1 |
| squalene synthase | g1629 | *Ganoderma lucidum* | e^-161^ | ABF57213 |
| squalene monooxygenase/squalene epoxidase | g1801 | *Polyporus arcularius* | e^-167^ | TFK94486.1 |
| Squalene synthase-domain-containing protein | g10087 | *Thelephora terrestris* | 3e^-076^ | KAF9781669.1 |
| Alpha-muurolene synthase | g3648  g8354 | *Pleurotus ostreatus* | e^-137^  2e^-073^ | XP_036627630.1  XP_036627630.1 |
| 11-oxo-β-amyrin 30-oxidase | g4129  g4146 | *Inonotus obliquus* | 0  0 | QEP49721.1  QEP49721.1 |
| lanosterol synthase | g2189 | *Ganoderma lucidum* | 0 | ADD60470 |
| Sesquiterpene synthase | g1456  g1445  g1440  g1454  g1451  g9044  g7464  g6603  g1458  g6614  g6608  g6631  g6636  g6629  g1439  g6607  g6613  g3395 | *Agrocybe aegerita* | 3e^-69^  2e^-66^  1e^-65^  3e^-64^  1e^-61^  6e^-59^  4e^-43^  3e^-35^  4e^-33^  4e^-32^  6e^-30^  9e^-29^  5e^-26^  1e^-24^  2e^-24^  8e^-24^  5e^-19^  9e^-10^ | QGA30884.1  QGA30884.1  QGA30884.1  QGA30884.1  QGA30884.1  QGA30884.1  QGA30884.1  QGA30884.1  QGA30884.1  QGA30884.1  QGA30884.1  QGA30884.1  QGA30884.1  QGA30884.1  QGA30884.1  QGA30884.1  QGA30884.1  QGA30884.1 |
| polyketide synthase | g7818  g3722  g1589  g3761 | *Lentinula edodes* | 1e-068  2e^-051^  6e^-035^  1e^-034^ | GAW05504.1  GAW05504.1  GAW05504.1  GAW05504.1 |
| non-ribosomal peptide synthase | g2559  g838  g6895  g386  g593 | *Coprinus cinereus* | 0  e^-156^  9e^-046^  2e^-041^  7e^-036^ | A8NS27.3  A8NS27.3  A8NS27.3  A8NS27.3  A8NS27.3 |

**Table S14 P450 related to betulinic acid and inotodiol synthesis in *Inonotus obliquus* genome**

| **Top Annotation** | **Gene ID** | **Species** | **E -Value** | **Accession Number** | **CYP Family** |
| --- | --- | --- | --- | --- | --- |
| CYP97B62 | g5553  g3172  g6779  g1343 | *Ricinus communis* | 2e^-038^  7e^-034^  1e^-033^  7e^-031^ | XP_002520583.1 | CYP63  --  --  -- |
| CYP86A182 | g7106  g3231  g1560  g4146 | *Quercus suber* | 6e^-026^  3e^-025^  2e^-023^  2e^-023^ | ABZ80831.1 | CYP5032  CYP63  --  -- |
| CYP89S1 | g6587  g8846  g6999 | *Capsicum chinense* | 4e^-035^  3e^-027^  1e^-026^ | PHU23374.1 | CYP5148  CYP5037  CYP5144 |
| CYP90B3 | g10443  g5147  g9278  g2933 | *Solanum lycopersicum* | 1e^-017^  7e^-017^  3e^-016^  1e^-015^ | NP_190635.1 | CYP512  CYP51  CYP51  CYP512 |
| CYP724B2 | g1217  g9662 | *Solanum lycopersicum* | 3e^-014^  2e^-012^ | BAF41218.1 | CYP620  CYP5150 |

CYP97B62, CYP86A182, and CYP89S1 related to betulinic acid synthsis; CYP90B3 and CYP724B2 related to inotodiol synthesis; -- means no relevant family classification.

**Table S15** **Identification of cytochrome P450 genes in *I. obliquus* genome.**

| **Query** | **Hit type** | **PSSM-ID** | **From** | **To** | **E-Value** | **Bitscore** | **Accession** | **Short name** |
| --- | --- | --- | --- | --- | --- | --- | --- | --- |
| >g5238.t1 | specific | 410705 | 661 | 1074 | 3.21012E-91 | 296.944 | cd20612 | CYP_LDS-like_C |
| >g5241.t1 | specific | 410705 | 468 | 871 | 1.32404E-79 | 262.661 | cd20612 | CYP_LDS-like_C |
| >g5244.t1 | superfamily | 425388 | 626 | 1018 | 2.46681E-68 | 233.386 | cl41757 | cytochrome_P450 superfamily |
| >g5553.t1 | specific | 410686 | 52 | 502 | 0 | 516.34 | cd11063 | CYP52 |
| >g5771.t1 | superfamily | 425388 | 90 | 524 | 5.6411E-104 | 319.275 | cl41757 | cytochrome_P450 superfamily |
| >g6317.t1 | superfamily | 425388 | 101 | 471 | 2.3415E-167 | 477.895 | cl41757 | cytochrome_P450 superfamily |
| >g6432.t1 | specific | 410692 | 81 | 563 | 6.8424E-153 | 445.946 | cd11069 | CYP_FUM15-like |
| >g6587.t1 | specific | 410688 | 102 | 527 | 4.5779E-164 | 472.447 | cd11065 | CYP64-like |
| >g6662.t1 | specific | 410688 | 62 | 484 | 5.5165E-161 | 463.203 | cd11065 | CYP64-like |
| >g6779.t1 | specific | 410691 | 27 | 471 | 0 | 582.222 | cd11068 | CYP120A1 |
| >g1507.t1 | superfamily | 425388 | 555 | 976 | 1.9379E-113 | 356.965 | cl41757 | cytochrome_P450 superfamily |
| >g1518.t1 | specific | 410688 | 1 | 357 | 3.8801E-157 | 447.795 | cd11065 | CYP64-like |
| >g1523.t1 | superfamily | 425388 | 54 | 432 | 1.03977E-29 | 120.09 | cl41757 | cytochrome_P450 superfamily |
| >g1537.t1 | specific | 410688 | 138 | 563 | 3.1557E-167 | 482.463 | cd11065 | CYP64-like |
| >g1560.t1 | specific | 410651 | 20 | 403 | 2.29632E-62 | 206.211 | cd00302 | cytochrome_P450 |
| >g1620.t1 | superfamily | 425388 | 40 | 448 | 4.28095E-59 | 200.633 | cl41757 | cytochrome_P450 superfamily |
| >g1659.t1 | specific | 410688 | 64 | 491 | 0 | 531.383 | cd11065 | CYP64-like |
| >g1661.t1 | specific | 410688 | 71 | 494 | 0 | 522.138 | cd11065 | CYP64-like |
| >g1893.t1 | superfamily | 425388 | 97 | 508 | 7.85955E-77 | 248.398 | cl41757 | cytochrome_P450 superfamily |
| >g1999.t1 | specific | 410667 | 85 | 524 | 0 | 518.772 | cd11041 | CYP503A1-like |
| >g2000.t1 | specific | 410667 | 62 | 449 | 6.5968E-123 | 364.692 | cd11041 | CYP503A1-like |
| >g2001.t1 | specific | 410667 | 107 | 546 | 0 | 521.468 | cd11041 | CYP503A1-like |
| >g2003.t1 | specific | 410667 | 1 | 257 | 8.0981E-124 | 359.299 | cd11041 | CYP503A1-like |
| >g2004.t1 | specific | 410667 | 84 | 500 | 1.5489E-166 | 477.941 | cd11041 | CYP503A1-like |
| >g2006.t1 | specific | 410667 | 170 | 607 | 3.5085E-176 | 506.445 | cd11041 | CYP503A1-like |
| >g2007.t1 | superfamily | 425388 | 12 | 97 | 2.45158E-29 | 108.149 | cl41757 | cytochrome_P450 superfamily |
| >g2511.t1 | superfamily | 425388 | 633 | 1018 | 3.0234E-59 | 207.963 | cl41757 | cytochrome_P450 superfamily |
| >g4129.t1 | superfamily | 425388 | 128 | 561 | 1.0534E-124 | 373.914 | cl41757 | cytochrome_P450 superfamily |
| >g4130.t1 | superfamily | 425388 | 649 | 1012 | 8.37448E-41 | 155.837 | cl41757 | cytochrome_P450 superfamily |
| >g4133.t1 | superfamily | 425388 | 33 | 322 | 1.71019E-35 | 134.266 | cl41757 | cytochrome_P450 superfamily |
| >g4135.t1 | superfamily | 425388 | 1 | 272 | 5.36202E-30 | 117.317 | cl41757 | cytochrome_P450 superfamily |
| >g4139.t1 | specific | 410651 | 14 | 270 | 4.98712E-31 | 119.927 | cd00302 | cytochrome_P450 |
| >g4140.t1 | superfamily | 425388 | 67 | 440 | 1.76909E-44 | 160.845 | cl41757 | cytochrome_P450 superfamily |
| >g4146.t1 | specific | 410692 | 70 | 522 | 1.6203E-144 | 422.834 | cd11069 | CYP_FUM15-like |
| >g4503.t1 | specific | 410688 | 66 | 479 | 1.0308E-169 | 485.159 | cd11065 | CYP64-like |
| >g4864.t1 | specific | 410684 | 143 | 596 | 9.6147E-129 | 393.897 | cd11061 | CYP67-like |
| >g5147.t1 | specific | 410668 | 112 | 579 | 0 | 551.822 | cd11042 | CYP51-like |
| >g5207.t1 | specific | 410688 | 26 | 339 | 3.6507E-137 | 397.719 | cd11065 | CYP64-like |
| >g5214.t1 | specific | 410684 | 131 | 556 | 1.113E-123 | 370.785 | cd11061 | CYP67-like |
| >g3387.t1 | specific | 410692 | 90 | 552 | 7.1771E-166 | 478.688 | cd11069 | CYP_FUM15-like |
| >g3396.t1 | superfamily | 425388 | 125 | 482 | 2.0127E-120 | 360.046 | cl41757 | cytochrome_P450 superfamily |
| >g3667.t1 | superfamily | 425388 | 1 | 94 | 2.85204E-16 | 73.7658 | cl41757 | cytochrome_P450 superfamily |
| >g3765.t1 | superfamily | 425388 | 33 | 176 | 7.59432E-34 | 125.383 | cl41757 | cytochrome_P450 superfamily |
| >g3852.t1 | specific | 410684 | 707 | 1131 | 3.2708E-129 | 400.446 | cd11061 | CYP67-like |
| >g3863.t1 | specific | 410684 | 140 | 551 | 2.6918E-131 | 389.275 | cd11061 | CYP67-like |
| >g3864.t1 | specific | 410684 | 136 | 545 | 7.418E-146 | 428.565 | cd11061 | CYP67-like |
| >g745.t1 | specific | 410684 | 129 | 570 | 5.0506E-145 | 424.713 | cd11061 | CYP67-like |
| >g746.t1 | specific | 410684 | 140 | 567 | 3.461E-140 | 412.387 | cd11061 | CYP67-like |
| >g804.t1 | superfamily | 425388 | 1 | 300 | 2.5001E-105 | 314.901 | cl41757 | cytochrome_P450 superfamily |
| >g7867.t1 | superfamily | 425388 | 104 | 263 | 4.58574E-36 | 133.223 | cl41757 | cytochrome_P450 superfamily |
| >g7870.t1 | superfamily | 425388 | 10 | 51 | 1.89398E-05 | 40.3308 | cl41757 | cytochrome_P450 superfamily |
| >g7877.t1 | specific | 410688 | 73 | 497 | 0 | 591.859 | cd11065 | CYP64-like |
| >g7879.t1 | superfamily | 425388 | 68 | 274 | 2.50948E-78 | 244.024 | cl41757 | cytochrome_P450 superfamily |
| >g7881.t1 | superfamily | 425388 | 64 | 381 | 7.7542E-105 | 316.442 | cl41757 | cytochrome_P450 superfamily |
| >g7962.t1 | specific | 410693 | 67 | 489 | 6.1783E-112 | 338.535 | cd11070 | CYP56-like |
| >g8025.t1 | specific | 410684 | 79 | 538 | 5.3844E-155 | 458.996 | cd11061 | CYP67-like |
| >g8156.t1 | specific | 410667 | 97 | 532 | 1.1028E-162 | 469.466 | cd11041 | CYP503A1-like |
| >g8157.t1 | specific | 410667 | 91 | 506 | 3.307E-163 | 470.622 | cd11041 | CYP503A1-like |
| >g8158.t1 | specific | 410667 | 70 | 490 | 3.3953E-159 | 458.681 | cd11041 | CYP503A1-like |
| >g8179.t1 | specific | 410684 | 76 | 543 | 2.005E-156 | 452.833 | cd11061 | CYP67-like |
| >g7861.t1 | specific | 410688 | 53 | 459 | 4.7121E-167 | 478.225 | cd11065 | CYP64-like |
| >g7862.t1 | superfamily | 425388 | 728 | 1123 | 4.15266E-40 | 152.879 | cl41757 | cytochrome_P450 superfamily |
| >g6811.t1 | specific | 410688 | 66 | 490 | 0 | 528.686 | cd11065 | CYP64-like |
| >g6814.t1 | superfamily | 425388 | 52 | 217 | 1.35331E-16 | 77.7655 | cl41757 | cytochrome_P450 superfamily |
| >g6975.t1 | superfamily | 425388 | 2 | 38 | 1.68486E-07 | 44.1054 | cl41757 | cytochrome_P450 superfamily |
| >g6997.t1 | specific | 410688 | 7 | 437 | 2.4161E-168 | 479.766 | cd11065 | CYP64-like |
| >g6999.t1 | specific | 410688 | 96 | 528 | 6.9352E-160 | 462.047 | cd11065 | CYP64-like |
| >g7000.t1 | specific | 410688 | 148 | 549 | 2.6659E-135 | 400.03 | cd11065 | CYP64-like |
| >g7001.t1 | specific | 410688 | 1 | 368 | 3.4731E-136 | 395.407 | cd11065 | CYP64-like |
| >g7106.t1 | superfamily | 425388 | 82 | 530 | 7.6123E-120 | 363.513 | cl41757 | cytochrome_P450 superfamily |
| >g2931.t1 | superfamily | 425388 | 4 | 374 | 1.05168E-48 | 170.624 | cl41757 | cytochrome_P450 superfamily |
| >g2933.t1 | specific | 410667 | 3 | 442 | 3.2489E-170 | 484.874 | cd11041 | CYP503A1-like |
| >g3102.t1 | specific | 410688 | 2 | 309 | 5.2491E-142 | 408.119 | cd11065 | CYP64-like |
| >g3172.t1 | specific | 410691 | 27 | 471 | 0 | 601.096 | cd11068 | CYP120A1 |
| >g3231.t1 | superfamily | 425388 | 136 | 620 | 1.8623E-148 | 435.448 | cl41757 | cytochrome_P450 superfamily |
| >g1178.t1 | superfamily | 425388 | 63 | 363 | 5.968E-129 | 376.918 | cl41757 | cytochrome_P450 superfamily |
| >g1180.t1 | superfamily | 425388 | 111 | 374 | 5.23205E-88 | 272.144 | cl41757 | cytochrome_P450 superfamily |
| >g1181.t1 | superfamily | 425388 | 1 | 76 | 3.44709E-23 | 91.485 | cl41757 | cytochrome_P450 superfamily |
| >g1183.t1 | specific | 410688 | 47 | 371 | 1.5358E-150 | 432.387 | cd11065 | CYP64-like |
| >g1186.t1 | specific | 410688 | 5 | 319 | 3.8156E-146 | 418.905 | cd11065 | CYP64-like |
| >g1187.t1 | specific | 410688 | 2 | 309 | 6.9566E-139 | 400.03 | cd11065 | CYP64-like |
| >g1188.t1 | specific | 410688 | 5 | 319 | 6.7261E-143 | 410.815 | cd11065 | CYP64-like |
| >g1190.t1 | superfamily | 425388 | 3 | 287 | 2.1356E-126 | 367.673 | cl41757 | cytochrome_P450 superfamily |
| >g1191.t1 | superfamily | 425388 | 25 | 209 | 1.78934E-47 | 161.206 | cl41757 | cytochrome_P450 superfamily |
| >g1192.t1 | superfamily | 425388 | 1 | 69 | 8.64106E-34 | 118.834 | cl41757 | cytochrome_P450 superfamily |
| >g1193.t1 | superfamily | 425388 | 6 | 107 | 5.64249E-42 | 143.102 | cl41757 | cytochrome_P450 superfamily |
| >g1194.t1 | specific | 410688 | 84 | 409 | 2.9133E-147 | 425.453 | cd11065 | CYP64-like |
| >g1195.t1 | specific | 410688 | 39 | 353 | 6.9524E-134 | 389.244 | cd11065 | CYP64-like |
| >g1198.t1 | specific | 410688 | 11 | 319 | 1.2492E-141 | 407.734 | cd11065 | CYP64-like |
| >g1199.t1 | superfamily | 425388 | 51 | 284 | 6.1726E-111 | 328.383 | cl41757 | cytochrome_P450 superfamily |
| >g1204.t1 | superfamily | 425388 | 58 | 138 | 5.01617E-19 | 82.2402 | cl41757 | cytochrome_P450 superfamily |
| >g1207.t1 | superfamily | 425388 | 1 | 150 | 3.72377E-72 | 223.223 | cl41757 | cytochrome_P450 superfamily |
| >g1208.t1 | superfamily | 425388 | 1 | 109 | 4.86454E-28 | 106.123 | cl41757 | cytochrome_P450 superfamily |
| >g1211.t1 | superfamily | 425388 | 1 | 236 | 4.4117E-122 | 354.191 | cl41757 | cytochrome_P450 superfamily |
| >g1213.t1 | superfamily | 425388 | 41 | 287 | 7.0257E-107 | 317.597 | cl41757 | cytochrome_P450 superfamily |
| >g1217.t1 | specific | 410688 | 94 | 517 | 1.3372E-172 | 494.019 | cd11065 | CYP64-like |
| >g1219.t1 | superfamily | 425388 | 5 | 325 | 1.3374E-133 | 387.318 | cl41757 | cytochrome_P450 superfamily |
| >g1331.t1 | specific | 410688 | 74 | 495 | 0 | 530.227 | cd11065 | CYP64-like |
| >g1339.t1 | superfamily | 425388 | 78 | 198 | 5.31025E-24 | 99.2664 | cl41757 | cytochrome_P450 superfamily |
| >g1342.t1 | specific | 410692 | 69 | 516 | 6.8831E-166 | 477.147 | cd11069 | CYP_FUM15-like |
| >g1343.t1 | specific | 410692 | 68 | 520 | 1.6286E-160 | 463.665 | cd11069 | CYP_FUM15-like |
| >g9278.t1 | specific | 410668 | 88 | 548 | 0 | 560.682 | cd11042 | CYP51-like |
| >g9435.t1 | specific | 410667 | 80 | 520 | 5.2167E-179 | 510.297 | cd11041 | CYP503A1-like |
| >g9657.t1 | superfamily | 425388 | 459 | 516 | 8.81989E-08 | 54.5832 | cl41757 | cytochrome_P450 superfamily |
| >g9659.t1 | specific | 410692 | 63 | 524 | 1.0471E-161 | 471.754 | cd11069 | CYP_FUM15-like |
| >g9660.t1 | specific | 410692 | 63 | 524 | 1.9E-164 | 474.066 | cd11069 | CYP_FUM15-like |
| >g9661.t1 | specific | 410692 | 63 | 496 | 3.4364E-159 | 459.428 | cd11069 | CYP_FUM15-like |
| >g9662.t1 | specific | 410692 | 260 | 710 | 1.4592E-146 | 435.16 | cd11069 | CYP_FUM15-like |
| >g9740.t1 | superfamily | 425388 | 76 | 358 | 6.18141E-93 | 284.933 | cl41757 | cytochrome_P450 superfamily |
| >g9741.t1 | specific | 410692 | 187 | 645 | 1.4194E-148 | 437.857 | cd11069 | CYP_FUM15-like |
| >g9742.t1 | specific | 410692 | 104 | 524 | 4.1524E-153 | 445.176 | cd11069 | CYP_FUM15-like |
| >g9763.t1 | specific | 410692 | 83 | 543 | 4.584E-158 | 458.658 | cd11069 | CYP_FUM15-like |
| >g10029.t1 | specific | 410692 | 104 | 574 | 1.7923E-148 | 435.931 | cd11069 | CYP_FUM15-like |
| >g10216.t1 | specific | 410692 | 64 | 524 | 8.2936E-154 | 449.413 | cd11069 | CYP_FUM15-like |
| >g10442.t1 | specific | 410667 | 80 | 519 | 0 | 529.943 | cd11041 | CYP503A1-like |
| >g10443.t1 | specific | 410667 | 88 | 519 | 4.1984E-179 | 511.453 | cd11041 | CYP503A1-like |
| >g10763.t1 | superfamily | 425388 | 68 | 514 | 2.5006E-126 | 376.225 | cl41757 | cytochrome_P450 superfamily |
| >g8459.t1 | specific | 410692 | 80 | 568 | 4.0668E-143 | 421.293 | cd11069 | CYP_FUM15-like |
| >g8615.t1 | specific | 410688 | 66 | 481 | 0 | 514.434 | cd11065 | CYP64-like |
| >g8626.t1 | specific | 410688 | 75 | 521 | 2.0956E-172 | 495.559 | cd11065 | CYP64-like |
| >g8837.t1 | specific | 410688 | 66 | 490 | 0 | 608.038 | cd11065 | CYP64-like |
| >g8846.t1 | specific | 410688 | 70 | 495 | 0 | 589.163 | cd11065 | CYP64-like |
| >g8866.t1 | specific | 410651 | 71 | 445 | 5.00939E-57 | 193.885 | cd00302 | cytochrome_P450 |
| >g8869.t1 | superfamily | 425388 | 59 | 485 | 1.38281E-57 | 196.817 | cl41757 | cytochrome_P450 superfamily |
| >g8885.t1 | superfamily | 425388 | 95 | 354 | 1.56032E-41 | 151.328 | cl41757 | cytochrome_P450 superfamily |
| >g8887.t1 | superfamily | 425388 | 7 | 199 | 2.22502E-27 | 106.9 | cl41757 | cytochrome_P450 superfamily |
| >g8900.t1 | superfamily | 425388 | 98 | 533 | 1.31755E-65 | 219.508 | cl41757 | cytochrome_P450 superfamily |
| >g2618.t1 | superfamily | 425388 | 57 | 475 | 7.71409E-64 | 212.96 | cl41757 | cytochrome_P450 superfamily |
| >g2619.t1 | superfamily | 425388 | 104 | 494 | 4.98069E-58 | 199.092 | cl41757 | cytochrome_P450 superfamily |
| >g2628.t1 | superfamily | 425388 | 54 | 185 | 4.60891E-07 | 49.2498 | cl41757 | cytochrome_P450 superfamily |
| >g11409.t1 | superfamily | 425388 | 76 | 564 | 2.3275E-105 | 323.097 | cl41757 | cytochrome_P450 superfamily |
| >g11425.t1 | superfamily | 425388 | 1 | 360 | 2.65609E-62 | 206.796 | cl41757 | cytochrome_P450 superfamily |
| >g9537.t1 | specific | 410688 | 66 | 490 | 0 | 528.686 | cd11065 | CYP64-like |
| >g9540.t1 | superfamily | 425388 | 52 | 217 | 1.35331E-16 | 77.7655 | cl41757 | cytochrome_P450 superfamily |
| >g4049.t1 | specific | 410684 | 76 | 533 | 1.0404E-154 | 448.596 | cd11061 | CYP67-like |
